# Clinical, radiographic and histomorphometric assessment of the effects of melatonin gel mixed with a xenograft in augmentation of the maxillary sinus: a randomized controlled clinical trial

**DOI:** 10.1186/s12903-026-08201-0

**Published:** 2026-04-16

**Authors:** Mohamed ElSholkamy, Dina M. Makawi, Sara M. ElKhateeb, Tasneem Soliman, Rehab A Soliman

**Affiliations:** 1 Department of Oral and Maxillofacial Surgery, Faculty of Dentistry, Suez Canal University, Ismailia, Egypt; 2 Department of Oral Biology, Faculty of Oral and Dental Medicine, Misr International University, Cairo, Egypt; 3 Department of Basic Medical and Dental sciences, Collage of Dentistry, Gulf Medical University, Ajman, United Arab Emirates; 4 Department of Oral and Maxillofacial Radiology, Faculty of Dentistry, Ain Shams University, Cairo, Egypt; 5 Department of Basic Dental Sciences, College of Dentistry, Princess Nourah bint Abdulrahman University, Riyadh, Saudi Arabia; 6 Department of Oral and Maxillofacial Surgery, Faculty of Oral and Dental Medicine, Misr International University, Cairo, Egypt

**Keywords:** Melatonin, Xenograft, Sinus Augmentation, Dental Implants, Open sinus lifting

## Abstract

**Background:**

Insufficient bone volume is one of the major problems encountered in the rehabilitation of the edentulous posterior maxilla with an implant-supported prosthesis. Melatonin gel appears to have positive effects on improving dental implant osseointegration and bone defect repair. This study aimed to assess the efficacy of combining melatonin gel with a xenograft in promoting bone regeneration during maxillary sinus augmentation.

**Methods:**

A total of 16 patients with average residual alveolar bone height less than 4 mm indicated for maxillary sinus augmentation participated in this randomized controlled clinical trial. Patients were randomized into two groups: an intervention group (*n* = 8), which was treated with melatonin gel combined with a xenograft, and a control group (*n* = 8), which was treated with a xenograft alone. Cone beam computed tomography was carried out 8 months after surgery to assess the bone height gained, and histological evaluations were performed before implant installation to assess bone quality. All patients underwent clinical evaluations to assess healing progress and record any pain, swelling, or complications.

**Results:**

Radiographically, both groups demonstrated a considerable increase in vertical bone height eight months post-operatively; however, the control group exhibited a statistically greater increase (13.22±2.45). Histologically, the intervention group showed superior bone regeneration (44.5±5.8), with a significantly greater percentage of mature bone and overall improved bone quality than the control group.

**Conclusion:**

Melatonin may play a beneficial role in enhancing bone quality during maxillary sinus augmentation without increasing the risk of complications. These findings suggest its potential as a safe and effective adjunct in maxillary sinus augmentation procedures.

**Trial Registration:**

ClinicalTrials.gov, NCT06736821. Registered on December 8, 2024.

**Supplementary Information:**

The online version contains supplementary material available at 10.1186/s12903-026-08201-0.

## Background

In oral implantology, the posterior maxilla is considered one of the most challenging areas. Several anatomic limitations could be encountered after the loss of maxillary posterior teeth, such as deficient alveolar height and increased pneumatization of the maxillary sinus. In addition, the posterior maxillary bone is a medullary bone characterized by limited quantity and osseous density in relation to the premaxilla or mandible. The adjacent cortices of compact bone are generally very thin with minimal strength. This insufficient bone volume is one of the major problems encountered in the rehabilitation of the edentulous posterior maxilla with an implant-supported prosthesis. This issue arises from the reduction in alveolar bone height and the restricted amount of bone available for implant insertion due to the maxillary sinus [1]. Therefore, many techniques and materials have been proposed for the augmentation of atrophic bone to receive an implant and improve masticatory function.

There are two methods used to perform sinus floor augmentation surgery. The first method is known as the osteotomy technique or closed technique, and it involves the use of osteotomes to carefully fracture the maxillary sinus floor. This method is indicated when the vertical bone height falls between 4 and 6 mm [2]. The second technique is the lateral window technique, which includes surgical exposure of the lateral wall of the maxillary sinus, exposing the Schneiderian membrane, which is elevated. The bone graft material is then carefully packed and placed on the sinus floor. The lateral window technique is preferred when the residual bone height is less than 4 mm [3, 4]. The type of grafting materials utilized and whether to place the implant simultaneously or later determine the main distinctions between the various operations when a lateral approach to the sinus is used [5].

Autogenous bone is believed to be the gold standard because of its high biocompatibility, osteoinductive, osteoconductive and good clinical outcomes [6]. However, autogenous bone tissue harvesting necessitates an additional surgical site, which increases the possibility of discomfort and morbidity, especially when bone is taken from an extraoral location [7]. Various studies have been conducted to reach a conclusion on the ideal graft material with minimal risk of morbidity, whether through inventing a new substance or by enhancing existing grafts by adding bioactive materials. Accordingly, one of the greatest obstacles in clinical research has been the fabrication of bioactive surgical additives to control inflammation and hasten the healing process [8].

N-acetyl-5-methoxytryptamine, often known as melatonin, is a hormone that is mostly produced and released by the pineal gland [9]. This substance can promote angiogenesis during the healing of bone defects [10]. Furthermore, melatonin has antioxidant and direct free radical scavenging actions that can interfere with osteoclastic activity and inhibit bone resorption. Moreover, it can downregulate receptor activator of nuclear factor-B ligand (RANKL)-mediated osteoclast formation and activation. Conversely, melatonin contributes to the process of bone formation in a number of ways, including accelerating the differentiation of osteoblast cells by triggering the production of collagen type I, other bone matrix proteins, and bone markers such as osteocalcin, as well as shortening the time needed for cell differentiation from 21 to 12 days [11, 12].

Preclinical research has demonstrated that melatonin directly promotes the differentiation and growth of osteoblasts. It is therefore considered a particularly appealing chemical for use in bone healing, whether used alone or in conjunction with other growth factors. It not only increases bone mass but also promotes osteointegration and stimulates new bone development [13]. An in vitro study carried out in 2022 revealed the beneficial effects of melatonin on bone defect repair. On the basis of these results, the author suggested the superiority of melatonin for guided bone regeneration because of its versatility in reducing inflammation and enhancing angiogenesis and bone cell proliferation, which can improve its application in clinical settings [14]. ​ Clafshenkel et al. [15], in their study on rats, reported that the implantation of calcium melatonin scaffolds into critical-sized calvarial bone defects enhanced tissue infiltration and scaffold biodegradation after 3 and 6 months. It also enhances implant stability in the posterior maxilla with better osseointegration, according to a recent study [16]. In a vivo study investigating treatments for osteoradionecrosis (ORN) in a rat model found that both melatonin and ascorbic acid offer significant therapeutic and radioprotective benefits for bone healing after tooth extraction and radiation. The study illustrated that melatonin promotes bone repair through a dual mechanism: it stimulates new bone formation by increasing the expression of osteogenic markers like Bone Morphogenic Protein-2 (BMP-2), Osteonectin (ONC), and Alkaline Phosphatase (ALP), while simultaneously inhibiting bone degradation by decreasing the activity of the bone-resorbing marker TRAP (Tartrate-Resistant Acid Phosphatase). Moreover, the combination therapy of melatonin and ascorbic acid showed the most substantial reduction in tissue damage based on histological analysis, and exhibiting significant elevations in ALP and ONC levels. The results suggested that the combination of ascorbic acid and melatonin together enhanced bone healing by both stimulating new bone formation and protecting against radiation-induced degradation [17].

Research has widely explored melatonin’s role as a promising biomimetic agent in bone regeneration, particularly in procedures like maxillary sinus augmentation. The mechanism primarily involves a dual action on bone cells and powerful antioxidant properties. Melatonin acts to significantly promote osteoblast differentiation and proliferation, enhancing the synthesis of key bone matrix proteins and accelerating the overall mineralization process as it was shown that preostoeblasts that were treated with melatonin matured into functional osteoblasts in just 12 days, which is nearly twice as fast as the untreated cells, which took 21 days to mature [18].

Recently, melatonin has emerged as a promising bioactive molecule with osteogenic, antioxidant, and anti-inflammatory properties. However, its application in maxillary sinus augmentation remains relatively unexplored, representing an innovative approach aimed at enhancing bone quality and regenerative outcomes. This hypothesis guided the present study to assess the impact of a melatonin–xenograft combination, compared with a xenograft alone, on bone development in maxillary sinus augmentation from clinical, radiological, and histological perspectives.

## Materials and methods

### Sample size calculation

The required sample size was calculated via PASS software, version 20 (NCSS, LLC, Kaysville, Utah, USA). A total of 16 participants (8 per group) were determined to be sufficient to detect a proportional difference in bone height gain following maxillary sinus augmentation between the intervention group (melatonin gel combined with xenograft) and the control group (xenograft only). The calculation was based on the effect size reported by Hallman et al. who evaluated bone regeneration following sinus floor elevation procedures [19]. Using a chi-square test for proportions and assuming a significance level (α) of 5% and a precision of 1%, this sample size was estimated to provide adequate statistical power to detect clinically meaningful differences between groups.

### Ethical consideration

This study was approved by the Research Ethics Committee of the Faculty of Dentistry, Suez Canal University, Ismailia, Egypt (IRB No. 710/2023) and was registered at ClinicalTrials.gov (NCT06736821; registration date: December 8, 2024). Written informed consent was obtained from all participants after the study objectives and procedures were fully explained.

### Study design, grouping, and setting

This was a parallel group randomized controlled clinical trial conducted at the outpatient clinic of the Faculty of Dentistry, Suez Canal University, Ismailia, Egypt. Sixteen patients with partially edentulous or free-ended saddle posterior maxillae requiring sinus augmentation were randomly assigned to two equal groups (*n* = 8) via a basic randomization sequence generated via an online tool (randomizer.org). In both groups, the sinus membrane was elevated via the lateral window technique. The intervention group received a combination of xenograft and melatonin gel, whereas the control group received only xenografts.

### Eligibility criteria

**Fig. 1:**
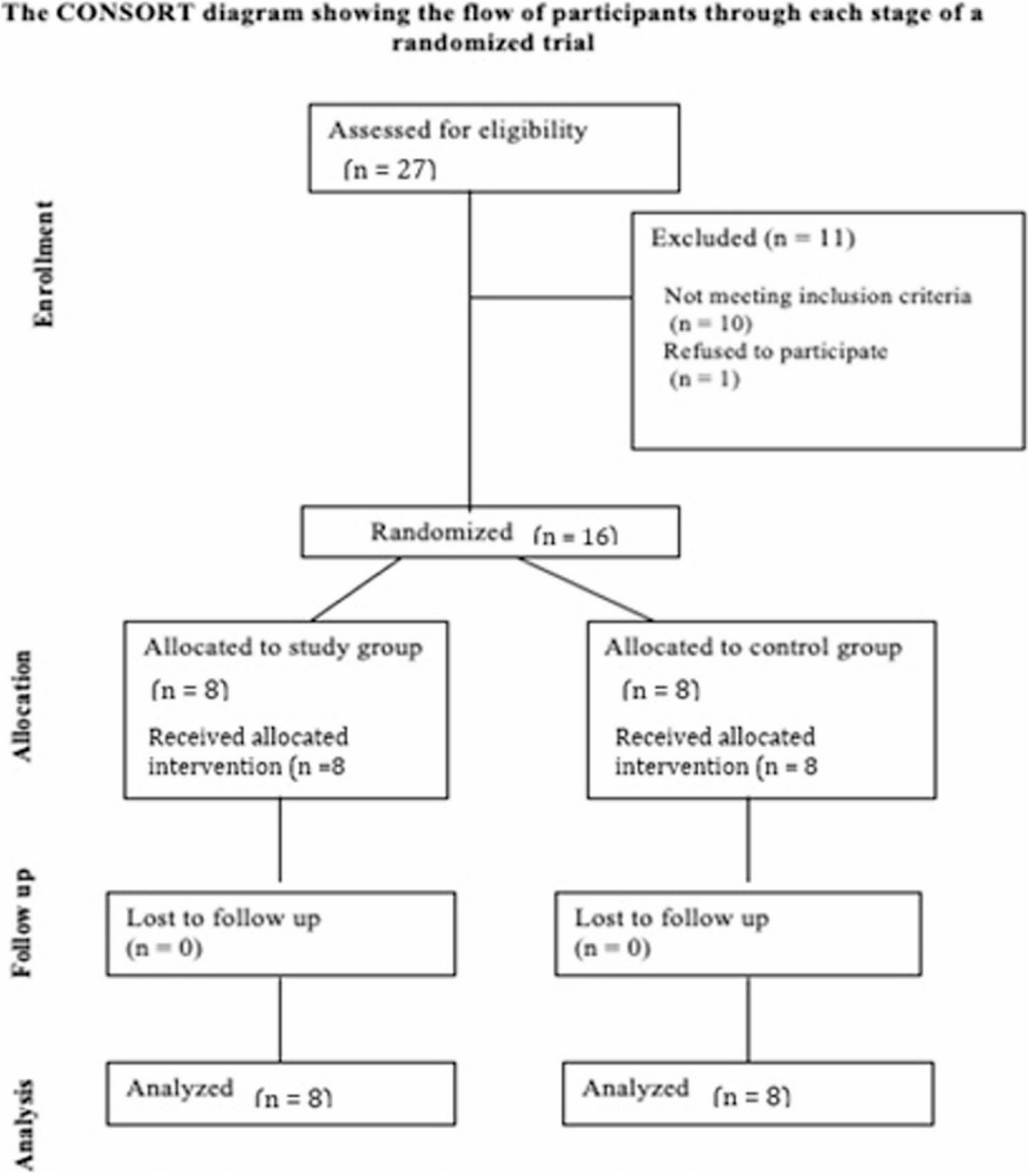
Flow of participants through the clinical trial. The CONSORT diagram illustrates the flow of participants at each stage of the randomized clinical trial. A total of 27 individuals were assessed for eligibility. Of these, 11 were excluded (10 did not meet the inclusion criteria and 1 refused to participate). Sixteen participants were randomized equally into two groups: the study group (*n* = 8) and the control group (*n* = 8). All participants received the allocated intervention, and no participants were lost to follow-up. All randomized subjects were included in the final analysis

Eligible participants had edentulous posterior maxillae with ≤ 4 mm alveolar bone height between the crest and sinus floor, requiring maxillary sinus lifting. Patients were excluded if they smoked more than 20 cigarettes per day [20], had periodontal disease affecting adjacent teeth, retained root fragments within the sinus, or presented with any form of maxillary sinus pathology. This study was conducted in accordance with the CONSORT guidelines for the reporting of randomized controlled trials (RCTs) (Fig. 1).

### Randomization and blinding

The participants meeting the eligibility criteria were randomly assigned to either the intervention group (xenograft mixed with melatonin gel) or the control group (xenograft alone) using a computer-generated random sequence (www.randomizer.org) with a 1:1 allocation ratio. Allocation concealment was achieved using sequentially numbered, opaque, sealed envelopes prepared by an independent coordinator. Allocation was concealed from the principal investigator until the day of surgery. Owing to the nature of the intervention, blinding of the surgeon was not feasible due to the distinguishable appearance of the materials. All procedures were performed by the same surgeon under a standardized protocol. However, the participants, outcome assessors, radiologist, histopathologists, and statistician were all blinded to the group assignments. For histological evaluation, each biopsy sample was labelled with a unique identification code before being sent to the histology laboratory. The histologist remained blinded to group allocation throughout the analysis to ensure unbiased assessment of all specimens.

### Melatonin gel preparation

A methylcellulose solution (1.5% w/v) was prepared by gradually adding the calculated amounts of the polymer (1.5 g methylcellulose, high viscosity 4000) while stirring using a magnetic stirrer RPM 50 to one third of the required amount (33 mL out of total 100 mL) of freshly prepared distilled water at 80 °C. The final volume was made by adding the remaining volume of water (approximately 67 mL), in which 150 mg of melatonin (MLN) was dispersed while stirring. The preparation was placed under vacuum to remove entrapped air prior to storage at 4 °C until required [21].

### Preoperative preparation

**Fig. 2:**
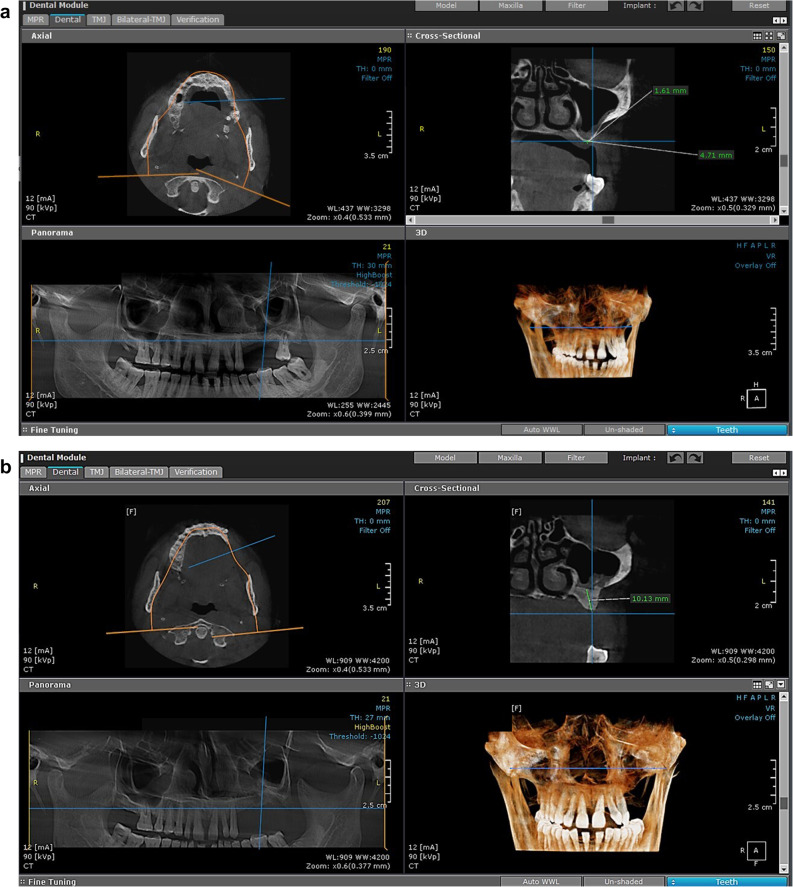
CBCT images showing changes in crestal bone height in the posterior left maxilla (intervention group). **A** Preoperative CBCT image showing the residual bone height (1.62 mm). **B** Postoperative CBCT image after 8 months, demonstrating the gained crestal bone height (10.13 mm)

Upon fulfilment of the eligibility criteria during clinical evaluation, all enrolled participants underwent preoperative radiographic assessment. CBCT scans were obtained for all patients using the same CBCT unit (Papaya 3D Plus, Genoray Co., Korea) with standardized exposure parameters (90 kV, 12 mA, exposure time 14.5 s). Residual bone height was measured on coronal CBCT images reconstructed perpendicular to the alveolar ridge at the planned implant site. Measurements were taken along a vertical line aligned with the long axis of the intended implant, from the most coronal point of the alveolar crest to the lowest point of the maxillary sinus floor. (Fig. 2A). All measurements were obtained using the digital measurement tools provided by the CBCT software [OnDemand3D™ dental imaging software (Cybermed Inc., Seoul, South Korea)]. To ensure standardization and reproducibility, the same image orientation, slice thickness, and measurement protocol were applied for all cases. Measurements were recorded in millimeters and tabulated for both study groups for statistical analysis. The measurement of residual bone height in both groups was tabulated (Table 1).

**Table 1:** Measurements of the preoperative residual bone height

Residual bone height (Intervention group)	Residual bone height (Control group)
3	3
4	2
2.5	3.5
2	2.5
3.6	2
1.5	3.4
3.75	2.5
4	2

### Surgical procedure

All surgical procedures were performed by a single experienced oral surgeon (R.A.S), who has substantial training in sinus floor elevation techniques. All surgical procedures were carried out under local anaesthesia with articaine 4% and adrenaline 1:100,000. A full thickness mucoperiosteal flap was reflected after a crestal incision was made under local anaesthesia to reveal the lateral wall of the maxillary sinus. Maxillary sinus floor elevation was subsequently carried out via the lateral window technique. A bone window was outlined using a no. 8 diamond bur mounted on a straight hand piece with copious irrigation (sterile saline solution), with careful precautions taken to avoid penetrating the sinus membrane. The process of bone removal was performed through the cortical bone to reach the membrane without perforation, and complete osteotomy was performed up to the Schneiderian membrane along the edge of the osseous window. The Schneiderian membrane was then carefully raised to the appropriate height, followed by graft application.

In both groups, sinus augmentation was performed using the same xenograft material to eliminate variability related to graft composition. The material used was deproteinized bovine bone mineral (DBBM), which is commercially known as RE-BONE^®^ (UBGEN S.r.l., Italy) and is 0.25–1.0 mm in size. On the basis of the patient’s allocation, patients were divided into either an intervention (xenograft + melatonin) or a control group (xenograft), which were prepared as follows:

#### Intervention group

**Fig. 3:**
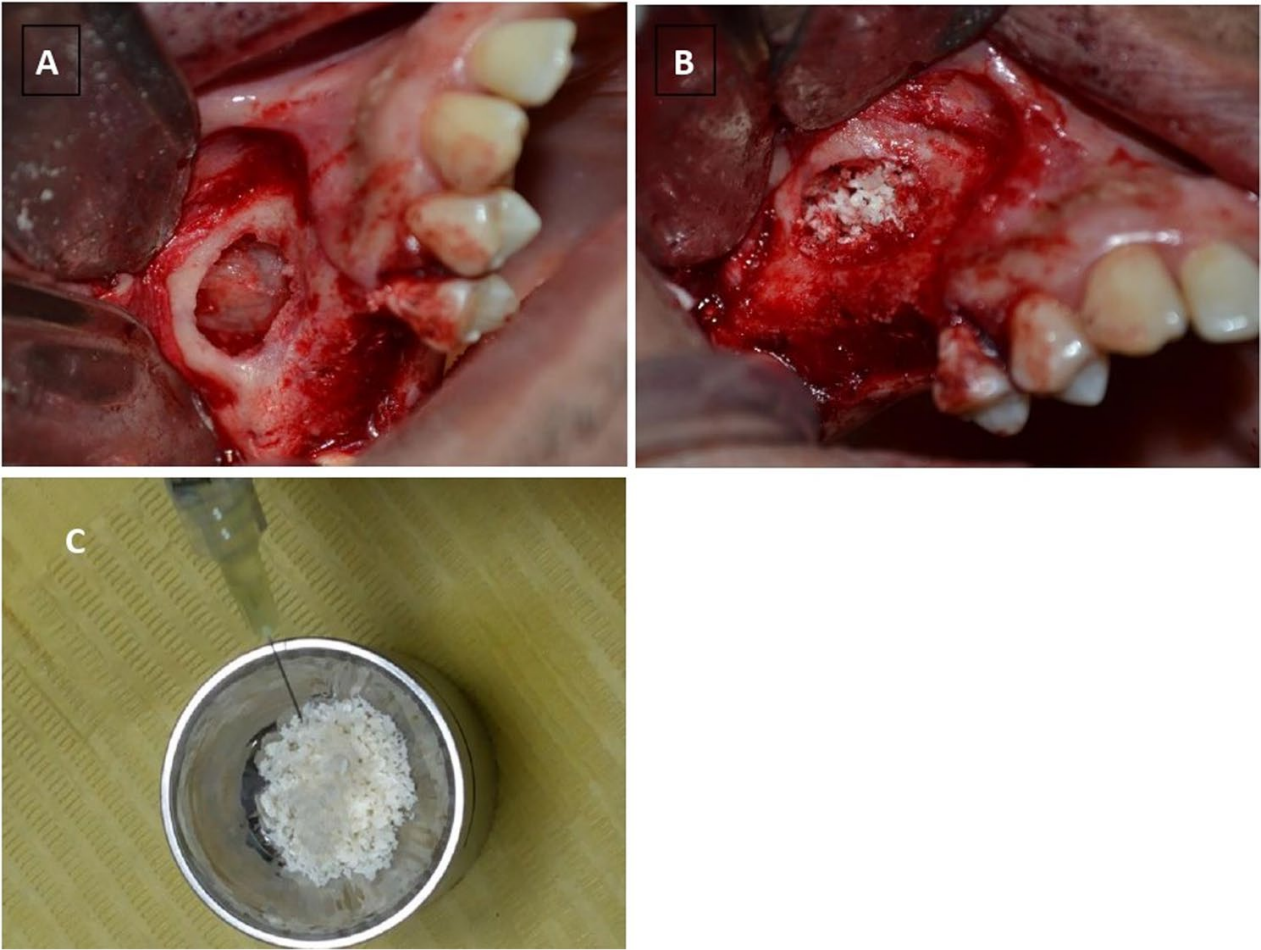
Intraoperative photographs of sinus membrane elevation and graft preparation. **A** Intraoperative view of the maxilla following sinus membrane elevation. **B** Placement of the xenograft material within the sinus cavity in the intervention group. **C** Preparation of the graft by mixing melatonin with the xenograft material prior to placement

Each 1 g of xenograft was mixed with 1 mL of melatonin gel containing 1.2 mg of melatonin. The gel was loaded into a sterile syringe to allow aseptic and uniform application over the xenograft granules. Mixing was performed at the surgical site under sterile conditions until complete coating and a homogeneous, mouldable consistency were obtained, ensuring the material was suitable for sinus grafting. Then the graft was packed and compacted against the walls of the sinus until a new available volume was created (Fig. 3A, B &C).

#### Control group

In the control group, the same amount of xenograft was mixed with 1 mL of sterile saline solution. Then the graft was pressed and compacted against the walls of the sinus until the newly generated volume was filled. In both groups, a collagen membrane was placed over the lateral window, and then the soft tissue flap was readapted and sutured via continuous and interrupted sutures (3–0 resorbable vicryl).

### Postoperative care

Postoperative instructions and medication, including Amoxicillin 875 mg + Clavulanic Acid 125 mg 2 tablets per day for one week (Hibiotic^®^, Amoun Pharmaceutical Co., Egypt), Ibuprofen 600 mg 2 tablets per day for one week (Brufen^®^, Abbott Laboratories, USA), and nasal spray Xylometazoline Hydrochloride 0.1%, 2–3 sprays every 12 h for one week (Otrivin^®^, Novartis Consumer Health, Switzerland), were prescribed for all patients [22, 23]. Suture removal and wound inspection were performed 7–10 days after surgery. All patients underwent clinical examinations every week for the first month and then three and six months after surgery.

### Postoperative radiographic evaluation

Eight months after surgery and before the second stage of surgery, postoperative CBCT images were obtained for each patient to evaluate the amount of bone height they had acquired. The same equipment and exposure parameters were used for CBCT. Image reconstruction was performed via Ondemand 3D software (cybermed, Inc., Korea).

**Fig. 4:**
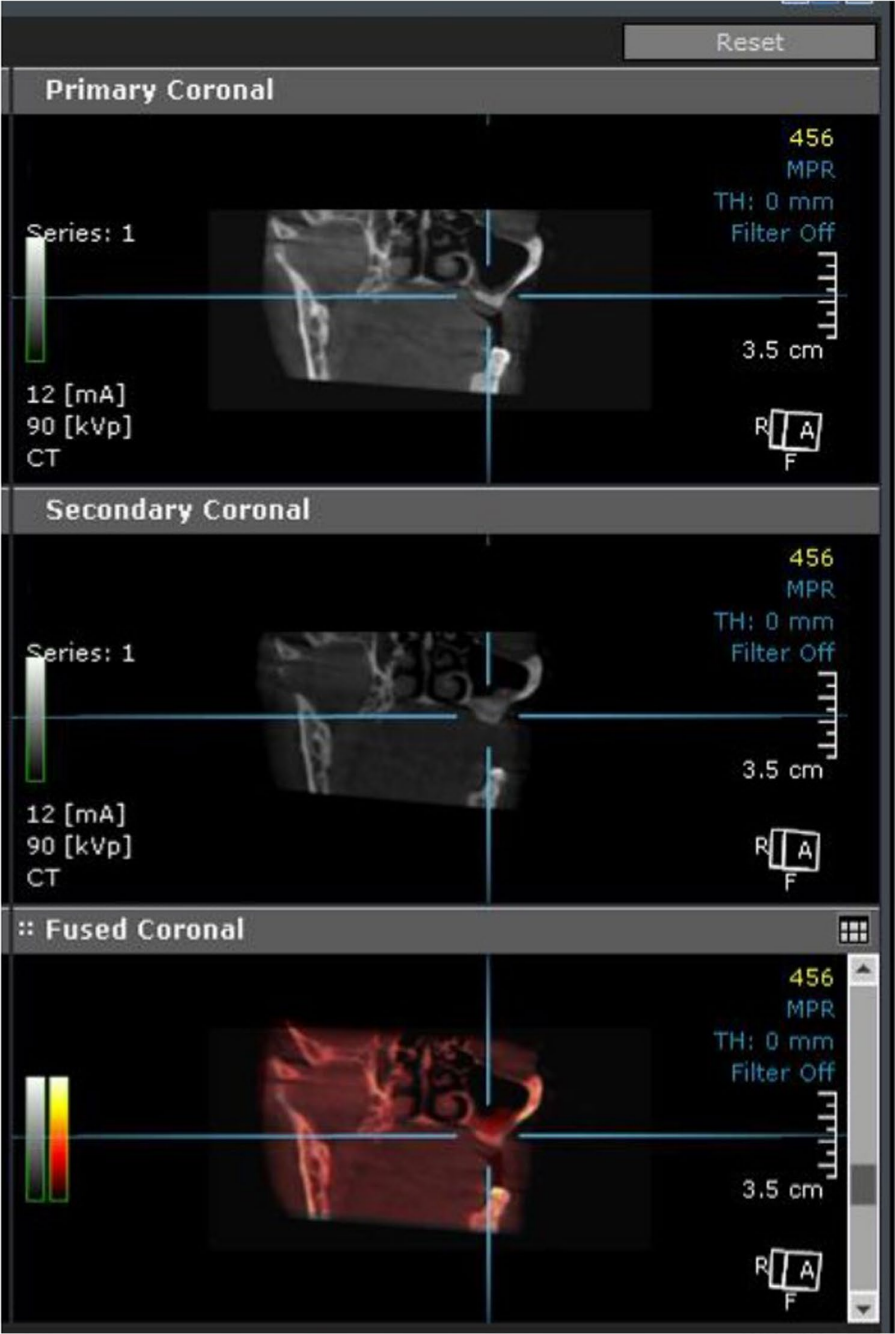
Superimposed CBCT images before and after sinus augmentation (intervention group). Fusion of preoperative and 8-month postoperative CBCT scans in the intervention group, illustrating the vertical bone gain following sinus augmentation using melatonin combined with a xenograft

Radiographic evaluation focused on measuring changes in bone height in each group. Using the ruler tool in CBCT software, the crestal bone height was measured in coronal section, and the distance from the crestal bone to the maxillary sinus floor was assessed (Fig. 2B). Fusion between preoperative and 8-month postoperative CBCT images was performed to evaluate the degree of bone height gain after augmentation, ensuring precise alignment of anatomical landmarks and enhancing the accuracy of linear measurements (Fig. 4). Ondemand 3D software with a millimeter scale was used to perform all measurements at the highest point at the new position of the sinus floor after the lifting procedure.

### Second-stage surgery

A second stage of surgery was carried out 8 months later to place the implant. A full thickness mucoperiosteal flap was reflected after a crestal incision was made under local anaesthesia. A trephine bur 3 mm in diameter (with an outer diameter of 3.0 mm, an inner diameter of 2.9 mm, and a length of 10 mm) was used instead of a solid twist drill for preparation; these biopsies were collected from the center region of the augmented maxilla for histological evaluation before implant placement. No extra defects were created. The drilling depth was planned from the CBCT to ensure that the biopsy contained newly formed and native bone. The implant was screwed via a ratchet wrench until the intraosseous portion of the implant was completely inserted into the bone. The cover screw was screwed into the fixture. Finally, the flap was returned to position and sutured via 3/0 vicryl suture.

**Fig. 5:**
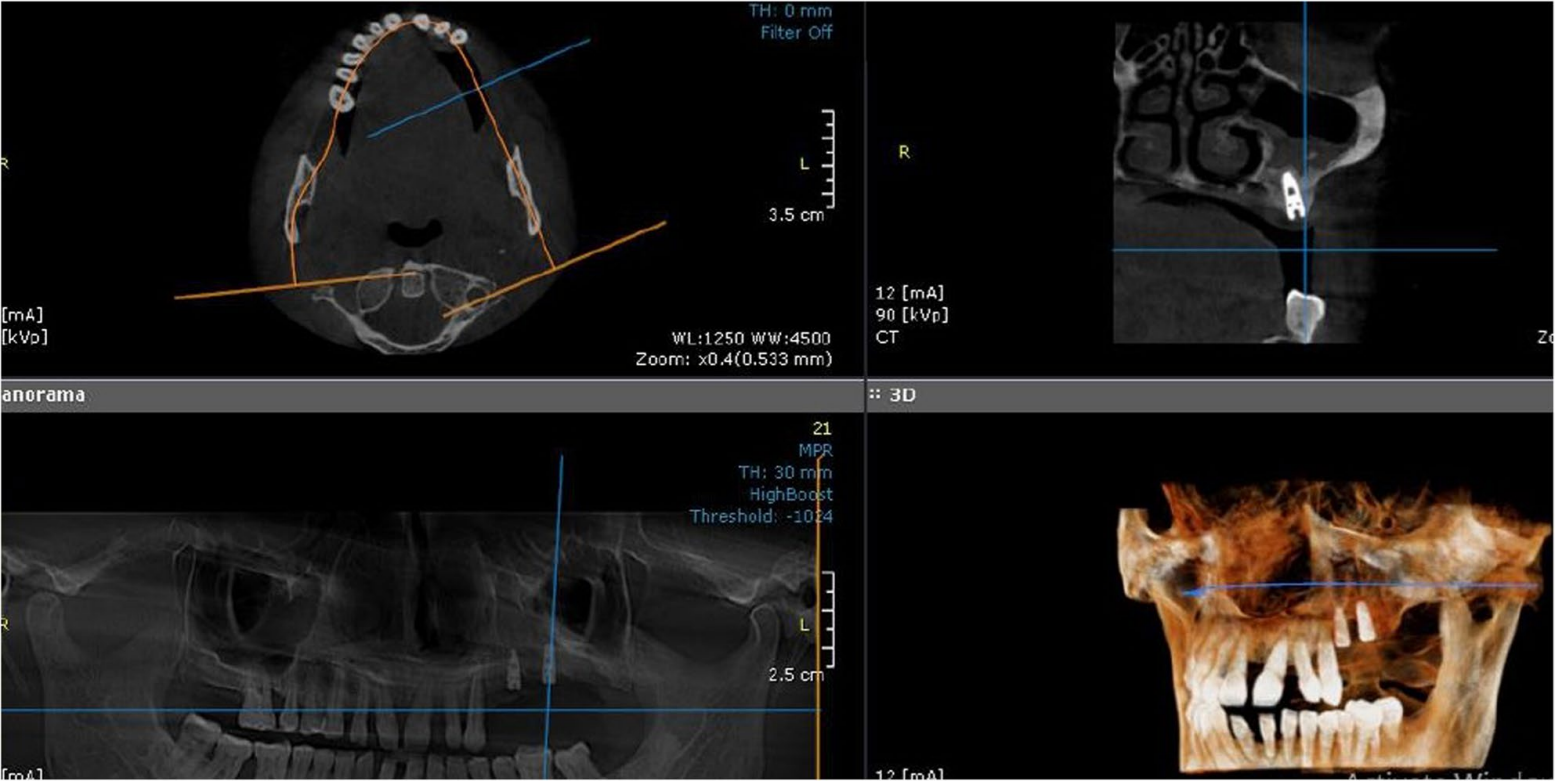
CBCT image after implant placement demonstrating osseointegration. Postoperative CBCT scan shows successful implant placement in the augmented maxillary area, with evidence of proper osseointegration and stable bone surrounding the implant

All the implants placed in the 16 elevated sinuses at the planned implant sites according to the preoperative work-up showed adequate primary stability during installation without intraoperative complications. A follow-up CBCT was performed after implant placement to ensure proper alignment (Fig. 5).

### Postoperative follow-up and assessment

#### Clinical assessment

All patients in both groups were examined for any signs or symptoms of postoperative pain, edema, sinus infection and/or dehiscence of the wound with local infection or inflammation one week, three months and eight months after surgery.

#### Radiographic assessment

CBCT imaging (Papaya 3D Plus, Genoray Co., Korea) with an exposure factor of 90 kV, a mA of 12 and an exposure time of 14.5 was used. The orientation beam was used to adjust the jawbone parallel to the reference surface. Measurements were carried out by a single calibrated examiner (S.M.E.) using OnDemand3D. To assess measurement reliability, intra-examiner agreement was evaluated by remeasuring 20% of the randomly selected scans after a two-week interval, resulting in an intra-class correlation coefficient (ICC) of 0.92. Consistent anatomical landmarks were used to ensure that the same region of the alveolar bone crest was measured in all scans. A linear measurement was performed on the multiplanar screen, and navigation was performed until an accurate view of the augmented sinus was observed on the reformatted panorama and sagittal cut. Using the tools from the machine software, a line was drawn from the new level of the sinus floor to the alveolar bone crest to measure the new alveolar bone height and width, which helps in choosing the proper implant dimensions (Fig. 2B).

#### Histopathological assessment

##### Specimen processing

After fixation in 10% buffered formalin, the samples were decalcified via a conventional protocol (soaking the tissue in the decalcifying agent at room temperature, typically with regular changes of the solution, with gentle agitation applied to speed up the process). 10% diluted formic acid was used for decalcification, as it is a weak organic acid that provides a balance between speed and tissue preservation. Its moderate action is less damaging to delicate cellular structures and nuclear staining than stronger acids, while still being faster than chelating agents and significantly faster than EDTA decalcification [24].

dehydrated through a graded series of ethanol concentrations, cleared in xylene, and embedded in paraffin. Each sample was labelled and longitudinally embedded to ensure standardized sectioning. Serial 4-µm-thick sections were cut in the longitudinal plane to ensure that each slide included both old and newly formed bone within the same field. The sections were stained with Hematoxylin and Eosin (H&E) and Masson’s trichrome (MT) for histological evaluation.

##### Histological analysis

**Fig. 6:**
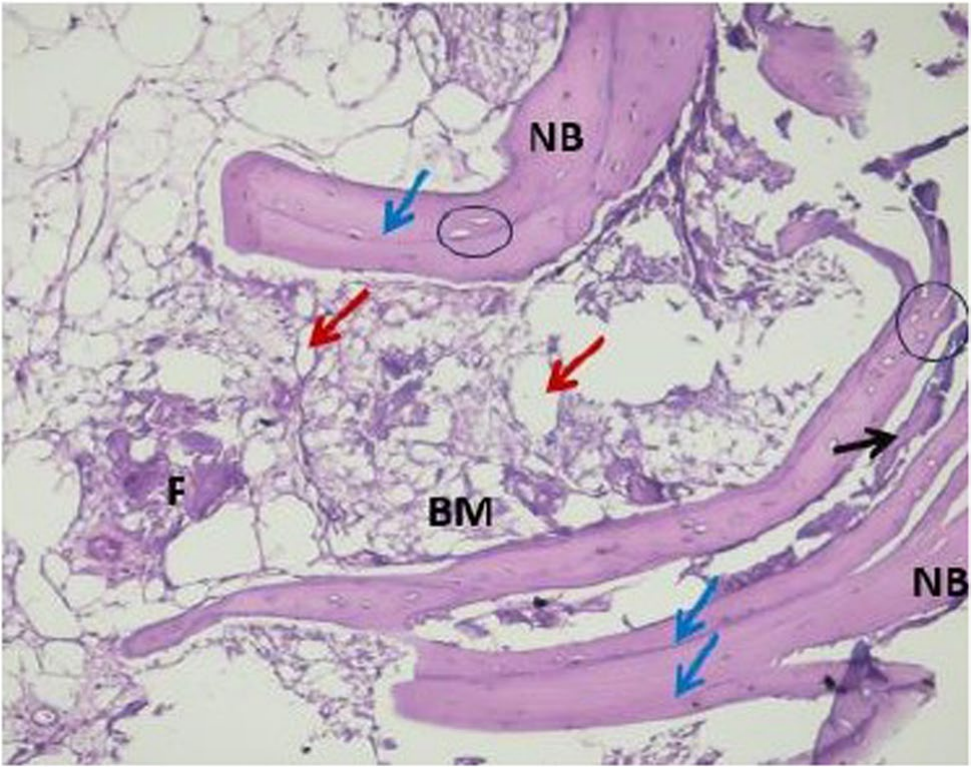
A photomicrograph of the control group xenograft core biopsy sample showing: thin bone trabeculae surrounded by wide irregular bone marrow cavities (BM), containing adipose tissue (red arrows). In between the bone trabeculae, fibrous connective tissue appears (F). Bone trabeculae appeared with resting line between the bone lamellae (blue arrows), with empty osteocytes lacunae (circle). Residual graft material is enclosed between the bone trabeculae (black arrow). (H&E x10 magnification)

**Fig. 7:**
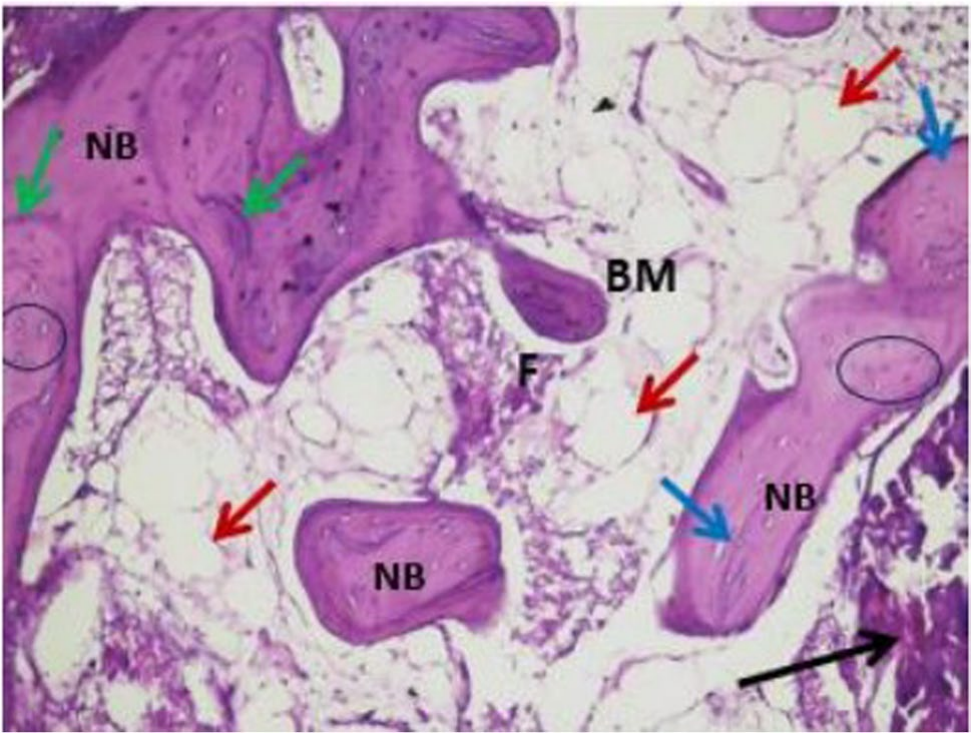
A photomicrograph of the intervention group xenograft +melatonin core biopsy sample showing: residual graft material (black arrow), variable sizes of bone marrow cavities (BM), areas of dense new bone trabeculae (NB), showing reversal lines (green arrows) denoting the active bone remodelling. In between the bone trabeculae, fibrous connective tissue appears (F) with intervening adipose tissue (red arrows). Bone trabeculae appeared with a resting line between the bone lamellae (blue arrows), with osteocytes in their lacunae (circle) (H&E x10 magnification)

**Fig. 8:**
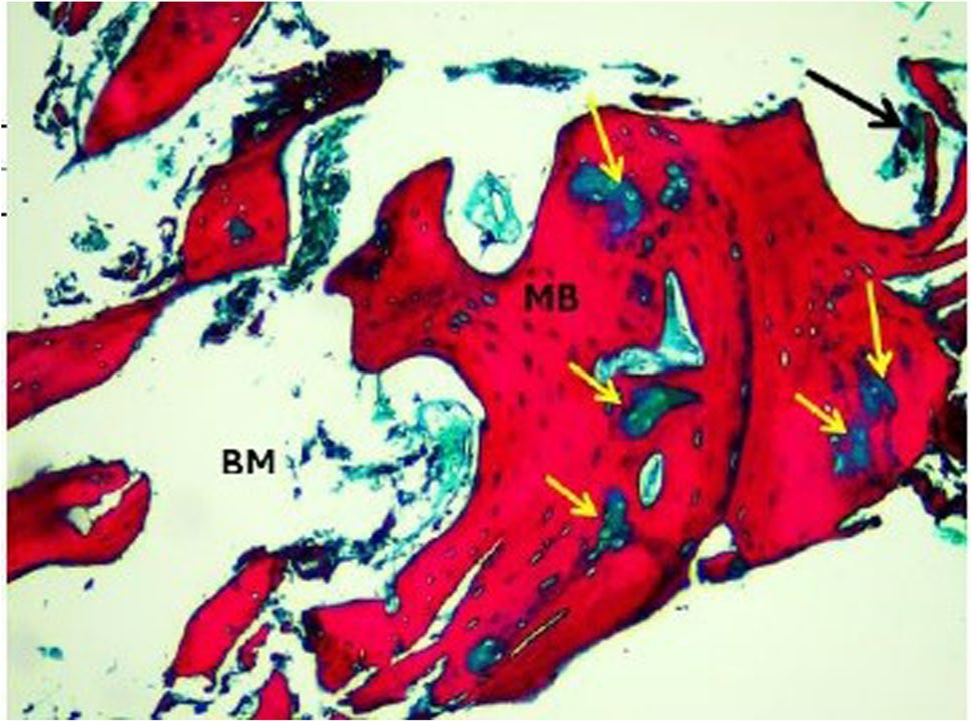
A photomicrograph of the control group. Xenograft core biopsy sample after a healing period of 8 months revealing the mineralized mature bone trabeculae stained red (MB), with multiple unmineralized areas (immature osteoid bone) stained blue (yellow arrows), residual graft material (black arrow), and bone marrow (BM). (Masson-trichrome, x10 magnification)

**Fig. 9:**
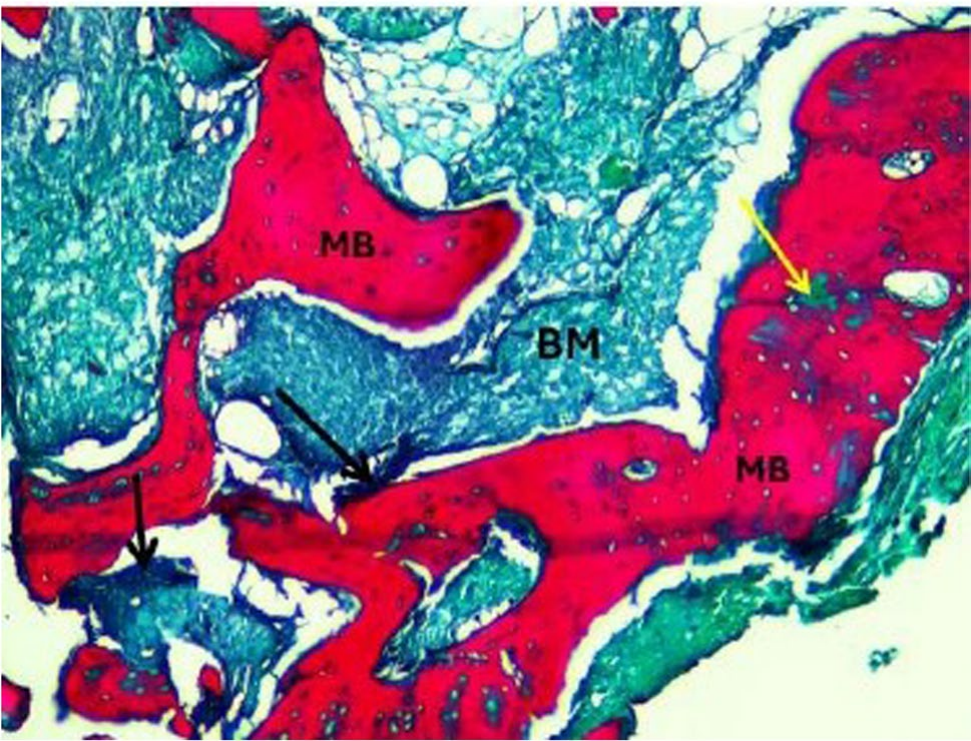
A photomicrograph of the intervention group. Xenograft + melatonin core biopsy sample after a healing period of 8 months revealing the mineralized mature bone trabeculae stained red (MB), with few unmineralized areas (immature osteoid bone) stained blue (yellow arrow), residual graft material (black arrows), and bone marrow (BM). (Masson-trichrome, x10 magnification)

Histopathological evaluation of the photomicrographs was performed via an HD camera (model No. XCAM1080PHB) mounted on a light microscope (SOPTOP EX20 biological microscope. China). Two types of stains were used. Decalcified H&E-stained sections were examined via an image analyser computer system applying Imagej 1.53e software (USA) (Figs. 6 and 7). The percentage area of new viable bone formed, and the percentage area of bone marrow were measured in five fields per case at X10 magnification power. In order to select the desired histological fields, the area of interest was selected according to the site of new bone formation, such as defect site. Meanwhile, areas that were not relevant to the measurement (e.g., surrounding soft tissue, non-bone elements) were excluded. The freehand selection tool in imageJ was used to contour the entire bone/defect area and use Edit> clear outside to mainly focus on the bone region. Decalcified Masson Trichrome-stained sections were also examined, and the area percentages of osteoid (blue colour) and mature bone were measured in five fields per case at X10 magnification (Figs. 8 and 9).

### Statistical analysis

Statistical analysis of the results was performed via SPSS software. The Shapiro‒Wilk test of normality was used to test the normality of all continuous variables. A paired t test was used to evaluate the statistical significance of differences in bone height before and after each treatment. The unpaired t test was used to evaluate the statistical significance of each parameter between the control group and the intervention group. P values ≤ 0.05 were considered statistically significant.

To evaluate the statistical robustness of the observed differences in the primary outcome, a post-hoc power analysis was performed using G*Power software (Version 3.1.9.4). Based on the observed means and standard deviations of mature bone% between the intervention group (43.64 ± 9.32) and the control group (26.63 ± 5.13), a large effect size (Cohen’s d = 2.26) was calculated. With a significance level of α = 0.05, the achieved statistical power was 98.7%, indicating that the study had sufficient power to detect the observed difference between groups.

To control for the increased risk of Type I errors associated with multiple comparisons across radiographic and histological parameters, a p-values were adjusted using the Benjamini–Hochberg False Discovery Rate (FDR) correction. A p-value (adjusted *q*-value) threshold of ˂ 0.05 was used to determine statistical significance for all primary and secondary outcomes.

## Results

Clinically, the area of interest was the posterior maxilla extending from the second premolar to the second molar. The clinical follow-up was performed immediately after surgery (one week after surgery) and at three and 8 months. Minimal swelling was observed at the surgical sites postoperatively and subsided by the end of the first week. During the following follow-up visits, all patients included in this study did not show any signs of inflammation or complications.

### Radiographic results

#### Crestal bone height measurement (mm)

**Fig. 10:**
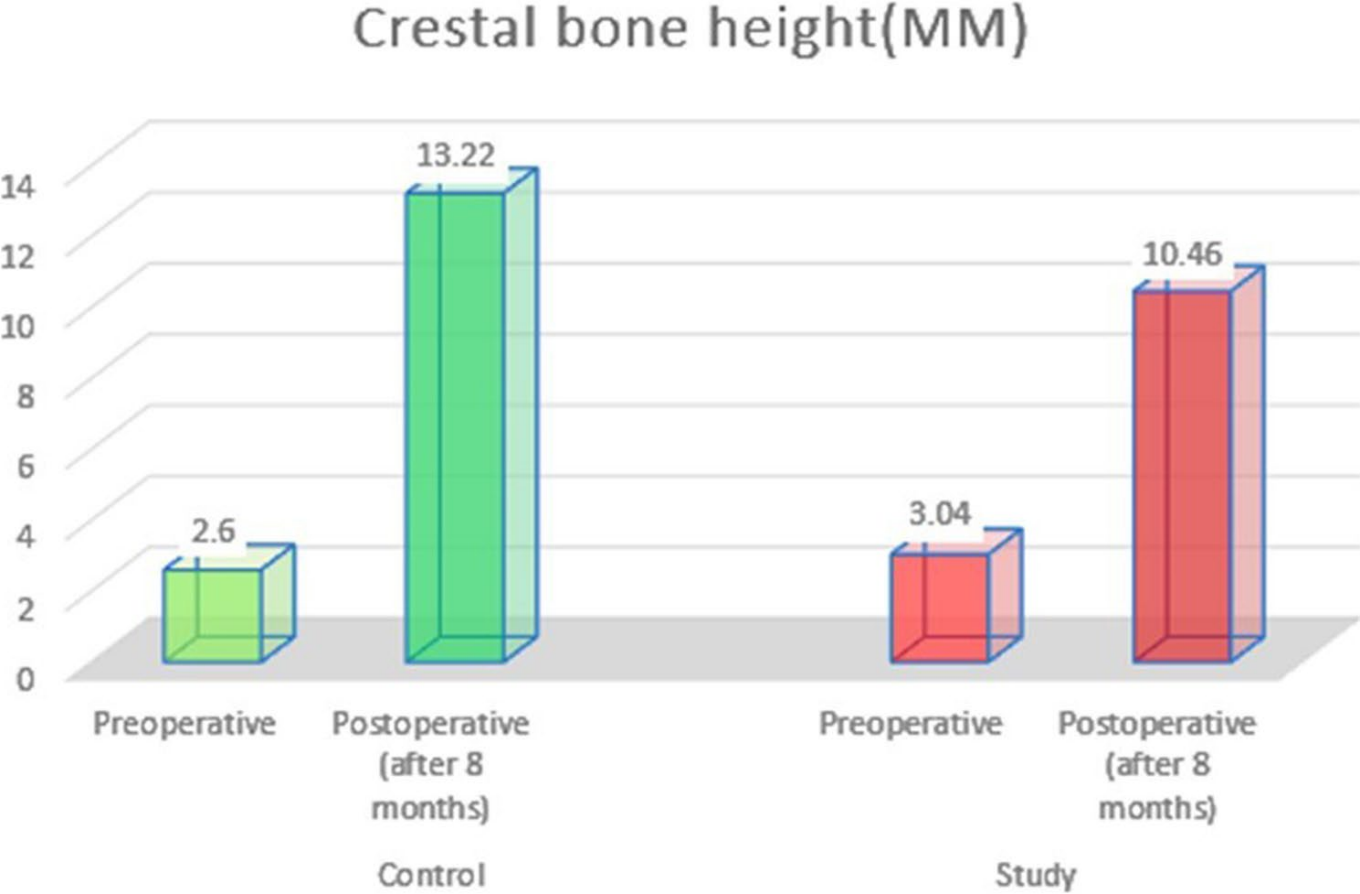
Column chart showing mean crestal bone height in all groups

Radiographically, the greatest mean crestal bone height (mm) was recorded in the control group postoperatively (after 8 months). A paired t test revealed that the postoperative height of the control group was significantly different from the preoperative height (*P* > 0.0001) (Table 2). A comparison of the postoperative crestal bone height of both groups via an unpaired t test revealed a significant difference between the control group and the intervention group (*P* = 0.00518) (Table 2) (Fig. 10).

**Table 2:** Comparison of crestal bone height (mm) between the two groups and the significance of the difference via paired t tests

	Control Group Preoperative	Intervention Group Preoperative	Control Group Postoperative (after 8 months)	Intervention Group Postoperative (after 8 months)
Mean	2.6	3.04	13.22	10.46
SD	0.62	0.95	2.45	0.95
Minimum	2	1.5	10	9
Maximum	3.5	4	16.8	12
P value	0.00001*		0.005*	

*Significant at *p* < 0.05

#### Adjusted comparison for baseline Crestal bone height

An adjusted comparison for baseline Crestal bone height using ANCOVA was performed, where the difference between the two groups remained statistically significant (*p* = 0.005). The adjusted mean Crestal bone height after 8 months was 13.41 ± 1.83 mm in the control group and 10.28 ± 1.83 mm in the intervention group. The adjusted mean difference was 3.128 mm (95% CI: 1.114 to 5.143), indicating that even after controlling for baseline values, the control group maintained significantly greater bone height. The effect size (Partial η² = 0.464) indicates a large effect, meaning that approximately 46.4% of the variance in Crestal bone height after 8 months can be attributed to group allocation after adjusting for baseline (Table 3).

**Table 3:** ANCOVA after adjusting for baseline Crestal bone height (mm) for the effect of groups on Crestal bone height (mm) after 8 months

	Control group (*n* = 8)	Intervention group (*n* = 8)	*p*-value	Mean difference (95% C.I)	Effect size Partial η²
Crestal bone height (mm) [after 8 months]	13.41 ± 1.83	10.28 ± 1.83	0.005^*^	3.128(1.114–5.143)	0.464

Data was expressed in Mean ± SD. *SD* Standard deviation Partial η²: Partial Eta Square

*CI* Confidence interval, *LL* Lower limit, *UL* Upper Limit

p: *p* value for comparing the two studied groups

*Statistically significant at *p* ≤ 0.05

#### Histological results

##### Area percentage of newly formed bone (%)

**Fig. 11:**
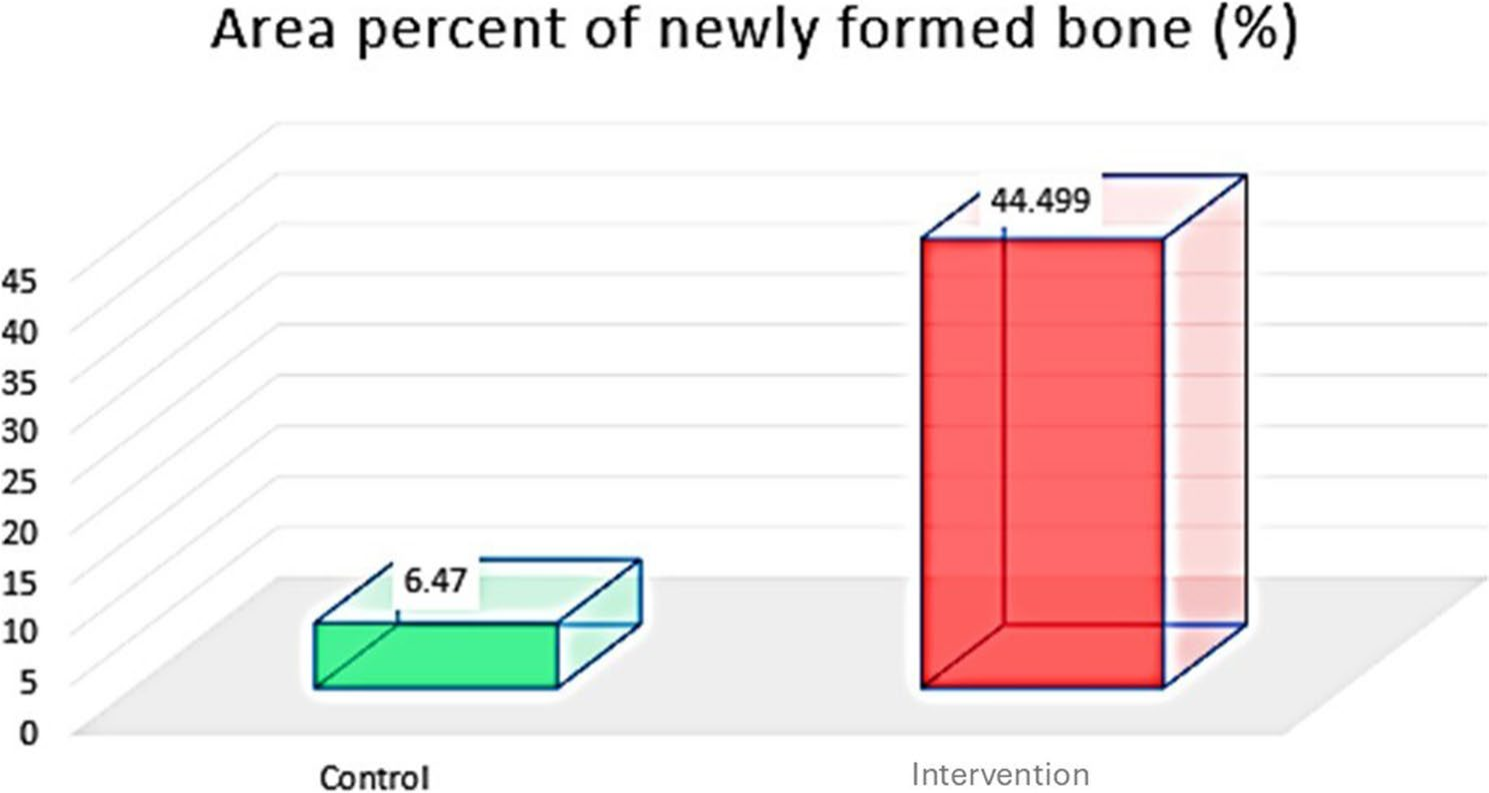
Column chart showing area percent of newly formed bone in both groups

Histological evaluation of the studied H&E-stained core biopsies from the control group revealed thin bone trabeculae surrounded by wide irregular bone marrow cavities with adipose and fibrous connective tissue. Moreover, resting lines appeared between the bone lamellae with empty osteocyte lacunae. Areas of residual graft material were noted between the bone trabeculae. In the intervention group, the bone appeared as dense lamellar trabeculae enclosing various sizes of bone marrow spaces containing loose connective tissue and adipose tissue. Fibrous connective tissue was found to intervene between the bone trabeculae. Moreover, the studied samples revealed incremental lines of bone; resting lines appeared between the bone lamellae with osteocytes in their lacunae. In addition, the bone reversal lines presented bone activity and remodelling with no signs of an inflammatory response (Figs. 6 and 7). The percentage area of newly formed bone was greater in the intervention group than in the control group. Unpaired t tests revealed significant differences between the two groups (*P* = 0.0029) (Table 4) (Fig. 11).

**Table 4:** Percent area of newly formed bone and bone marrow (area percentage %) in both groups and the significance of the difference according to unpaired t tests

	Control Group Newly Formed Bone	Intervention Group Newly Formed Bone	Control Group*Bone Marrow*	Intervention Group *Bone Marrow*
Mean	34.84	44.499	29.6	13.974
SD	6.47	5.798	1.9	4.814
Minimum	27	37.726	27	6.583
Maximum	44	52.167	32.5	22.045
P value	0.00291*		0.00001*	
Mean diff (95% C.I)	9.659 (3.0711 to 16.2469)		15.626 (11.7015 to 19.5505)	
Cohen’s d (95% C.I)	1.572 (0.451 to 2.694)		4.270 (2.495 to 6.045)	

*Significant at *p* < 0.05

##### Area percentage of bone marrow (%)

**Fig. 12:**
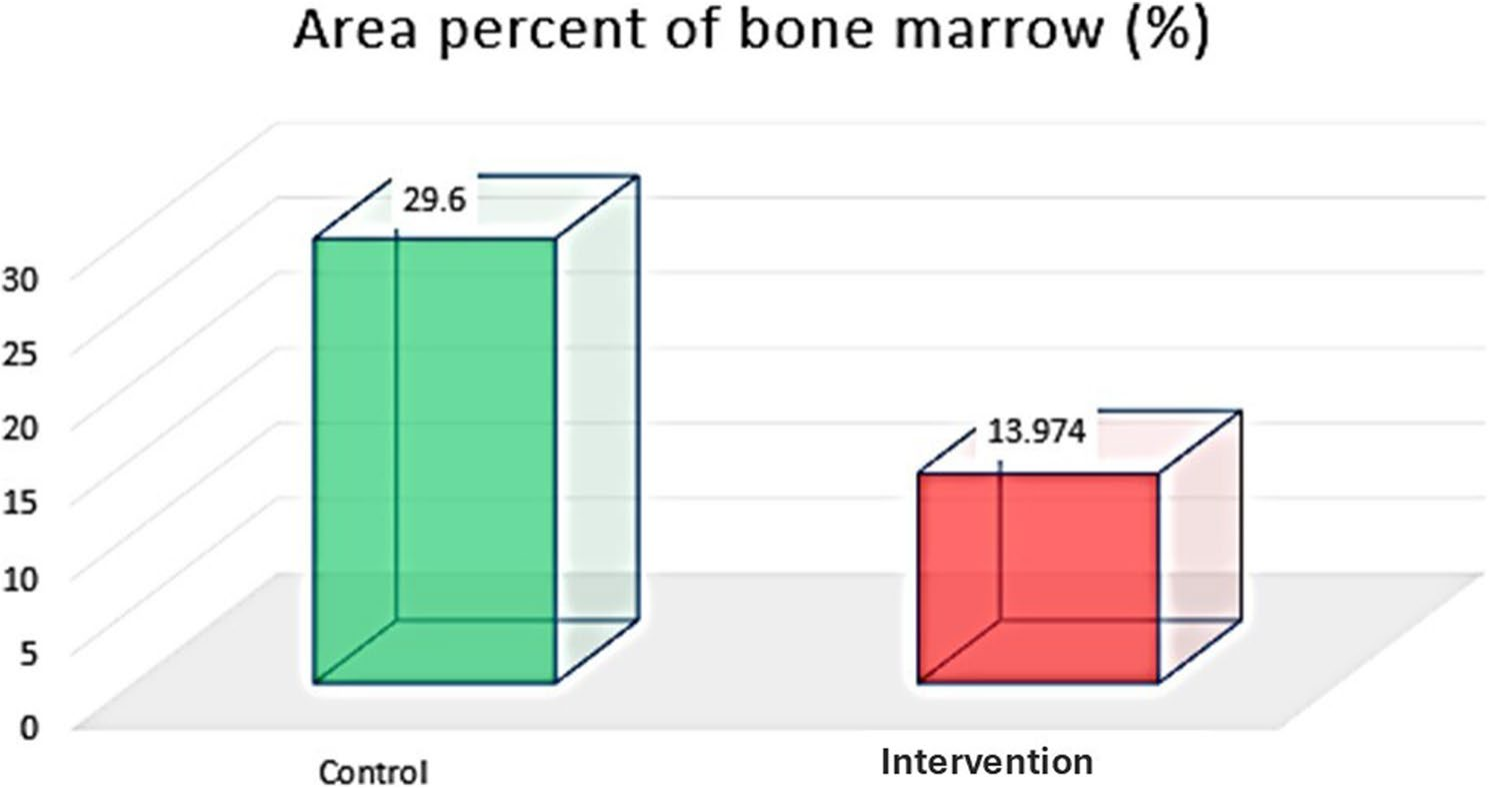
Column chart showing area percent of bone marrow in both groups

The percentage of bone marrow area was greater in the control group than in the intervention group. The unpaired t test revealed a significant difference between the two groups (*P* < 0.00001) (Table 4) (Fig. 12).

##### Area percentage of mature bone (%)

**Fig. 13:**
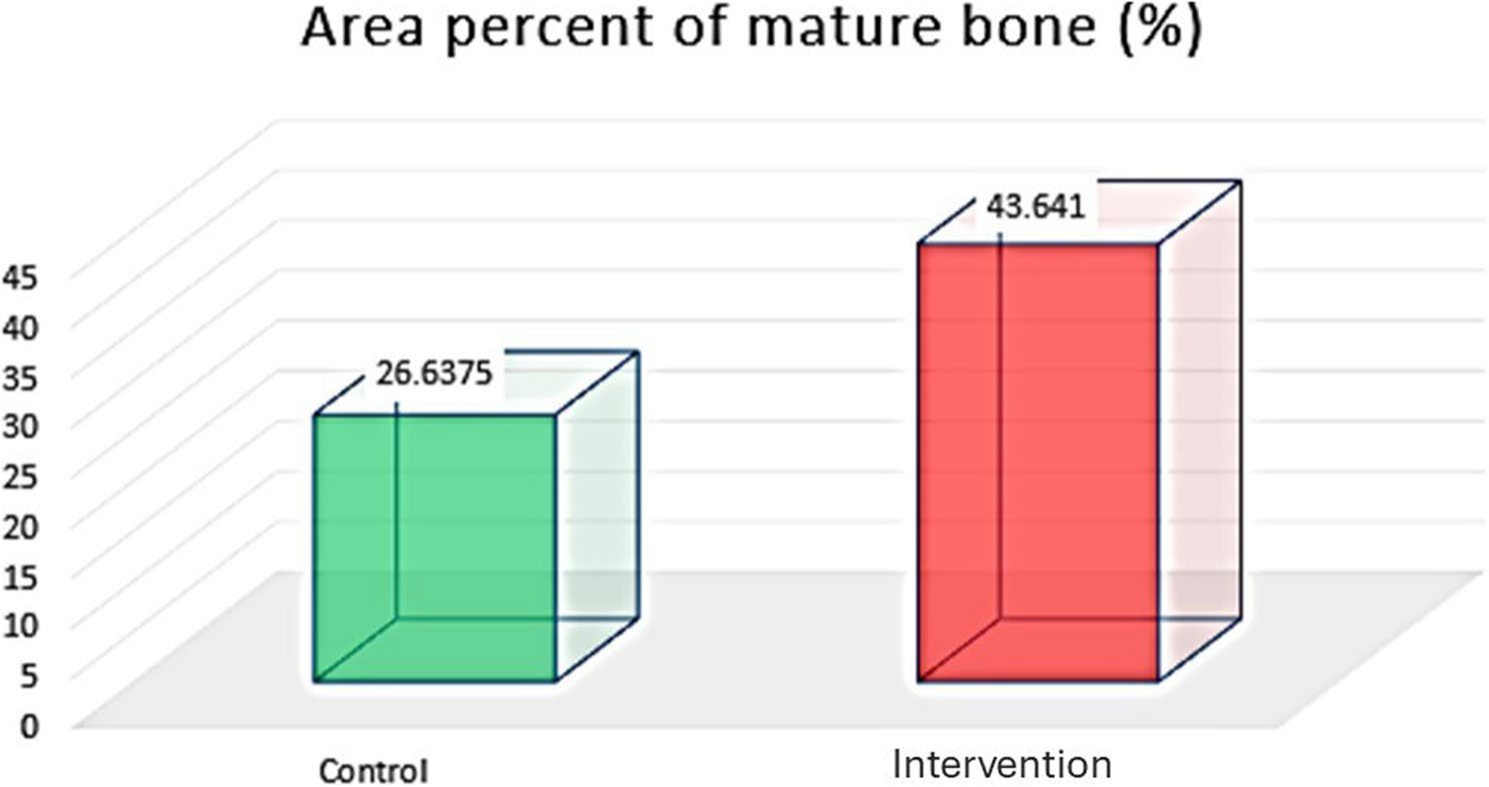
Column chart showing area percent of mature bone in both groups

Masson’s Trichrome stain was used to differentiate between the old and new bone, as the mature bone tissue stains red, and the uncalcified or osteoid tissue (immature) stains blue [25]. Histological evaluation of the studied core biopsies acquired from the control group via Masson’s trichrome revealed that mature mineralized bone was affected by multiple areas of osteoid unmineralized bone. Compared with those in the control group, the sections in the intervention group presented mature mineralized bone with fewer areas of osteoid unmineralized bone, indicating faster bone remodelling with melotnin addition. The mature bone showed normal lamellar organization (Figs. 8 and 9).The percentage of mature bone area was greater in the intervention group than in the control group. The unpaired t test revealed a significant difference between the two groups (*P* = 0.00024) (Table 5) (Fig. 13).

**Table 5:** Area percentage of mature and immature bone in both groups and the significance of the difference according to the unpaired t test (area percentage %)

	Control GroupMature Bone	Intervention GroupMature Bone	Control Group*Immature bone*	Intervention Group*Immature bone*
Mean	26.6375	43.641	24.45	7.693
SD	5.13%	9.32	4.00%	2.421
Minimum	17.8	33.387	20.50%	4.734
Maximum	35.8	59.051	32.50%	11.028
P value	0.00024*		0.00001*	
Mean diff (95% C.I)	17.004 (8.936 to 25.071)		16.757 (13.2115 to 20.3025)	
Cohen’s d (95% C.I)	2.260 (1.006 to 3.515)		5.069 (3.057 to 7.080)	

*Significant at *p* < 0.05

##### Area percentage of immature bone (%)

**Fig. 14:**
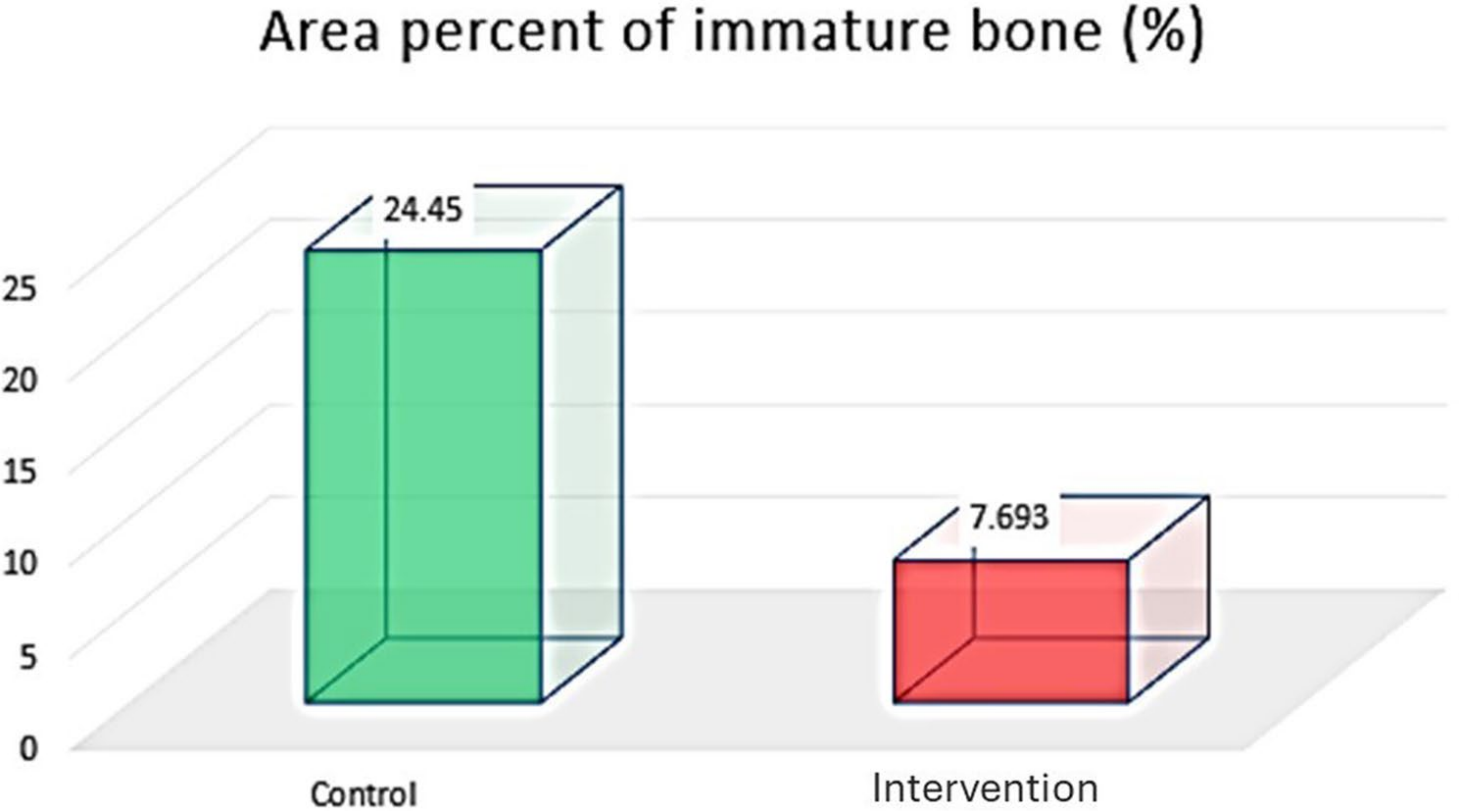
Column chart showing area percent of immature bone in both groups

The percentage of immature bone area was greater in the control group than in the intervention group. The unpaired t test revealed a significant difference between the two groups (*P* < 0.00001) (Table 5) (Fig. 14).

##### Area Percent of residual graft material (%)

**Fig. 15:**
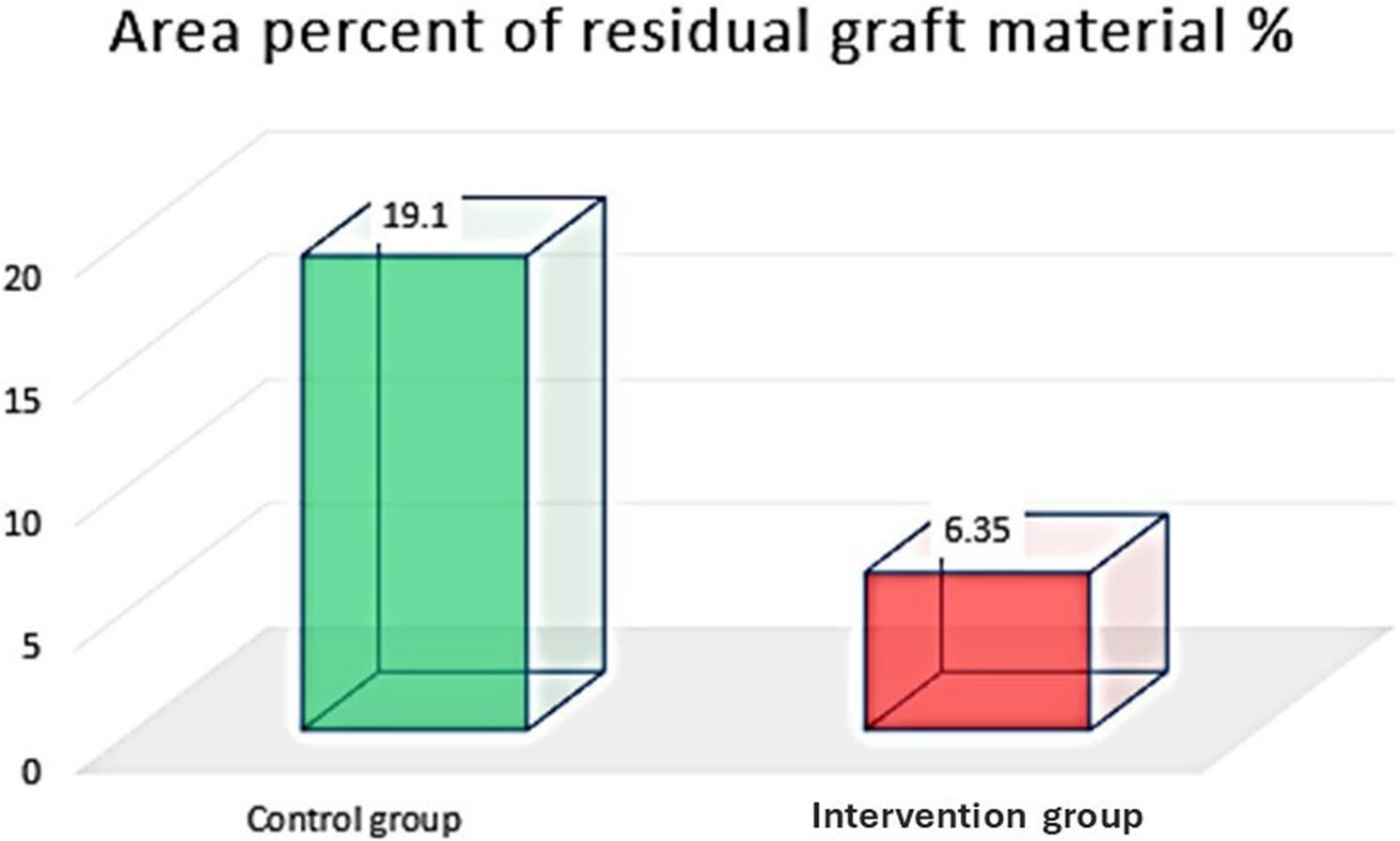
Column chart showing area percent of residual graft material in both groups

The area percent of graft material was higher in the control group than the study group. Unpaired T-test revealed significant differences between both groups (*P* < 0.0001) (Table 6) (Fig. 15).

**Table 6:** Area percent of residual graft material in both groups and significance of the difference using unpaired T-test (area percentage %)

	Control group	Study group
Mean﻿	19.1	6.35
SD	2.59	2.43
Minimum	15.8	3.25
Maximum	23.5	10.78
P-value	< 0.0001*	
Mean diff (95% C.I)	12.75 (10.057 to 15.443)	
Cohen’s d (95% C.I)	5.078 (3.064 to 7.092)	

*Significant at *p* < 0.05

#### Multiple comparison adjustments for radiographic and histological parameters

After adjustment for multiple comparisons using Benjamini–Hochberg adjusted p value and false Discovery Rate (FDR) method, all studied parameters remained statistically significant. Crestal bone height after 8 months showed a significant difference (adjusted *p* = 0.011). Similarly, newly formed bone demonstrated a statistically significant difference after adjustment (adjusted *p* = 0.009). Highly significant differences were observed for bone marrow, mature bone, immature bone, and residual graft material, with all adjusted p-values ≤ 0.0008.

These findings indicate that the observed differences are robust and remain significant even after controlling for potential type I error due to multiple testing (Table 7).

**Table 7:** Benjamini-hochberg adjusted *P*-value

	Original*p*-value	Exact *p*-value	*p*-value After adjusting by FDR
Crestal bone height (mm) [after 8 months]	0.011^*^	0.0106	0.011^*^
Newly Formed Bone	0.007^*^	0.0072	0.009^*^
Bone Marrow	< 0.001^*^	0.0001	0.0002^*^
Mature Bone	< 0.001^*^	0.0005	0.0008^*^
Immature bone	< 0.001^*^	0.0001	0.0002^*^
Residual graft material	< 0.001^*^	0.0001	0.0002^*^

*Statistically significant at *p *≤ 0.05

## Discussion

The aim of the current study was to evaluate the effectiveness of melatonin gel for bone regeneration in maxillary sinus augmentation. In the present study, 16 patients with an edentulous maxillary posterior region and insufficient vertical alveolar bone height were selected for the sinus augmentation procedure via the lateral window technique. The current study utilized xenograft as a control group without any additives. The selection of xenograft as a control was based on the consideration of xenograft as an ideal scaffold for new bone formation extensively applied in maxillary sinus floor augmentation [26]. The highest measured residual ridge height in this study was equal or less than 4 mm. Therefore, the lateral approach for sinus augmentation with delayed implant placement is the treatment of choice [2, 27–30].

While a 6-month re-entry is commonly reported in the literature, healing intervals following sinus augmentation vary widely, ranging from 6 to 9 months, particularly when xenograft materials are used because of their slow resorption and prolonged remodeling behavior [31, 32]. According to Filho et al. (2026), the 8-month healing period is a superior timeline for implant placement and specimen collection, as it demonstrated significantly increased bone formation (28.22 ± 6.29%) and a more advanced stage of maturation with organized collagen fibers compared to a shorter 4-month interval [31]. Accordingly, the 8-month healing interval adopted in the present study aligns with previously published evidence and was intended to ensure adequate graft maturation and stability. 

The novelty of this study lies in its histological investigation of the use of melatonin gel in the augmentation of the maxillary sinus via the lateral approach, which, to our knowledge, has not been previously evaluated in humans. However, this material has been previously investigated clinically in conjunction with dental implants [33] and was used in indirect sinus augmentation via the crestal approach [34].

The present study assessed the effect of melatonin in the maxillary sinus augmentation via histological examination of the degree of bone regeneration. The melatonin gel formulation used in this study followed the approach described by El-Gammal et al. (2016), who demonstrated the osteogenic potential of locally applied melatonin gels in promoting bone regeneration and implant osseointegration [16]. Although release kinetics were not analysed, these studies confirm the biological efficacy and clinical safety of this delivery form.

Histological evaluation is a more accurate assessment of bony neogeneration after sinus augmentation, as it allows direct visualization of all tissue components. The results of the present study revealed a statistically significant increase in newly formed bone and the maturation of bone by area percentage in the intervention group. These results agreed with other studies that demonstrated the efficacy of melatonin gel for bone regeneration. Dundar et al. [35] reported that local melatonin application increased osteogenesis in peri-implant bone tissues. Thus, they concluded that osteoblastogenesis might be directly induced by the local administration of melatonin during a surgical implant integration operation. Guardia et al. [36] utilized melatonin with dental implants in dogs and reported that it enhanced osteointegration, including the ratio of bone-to-implant contact, interthread bone, total peri-implant bone, and new bone production, two weeks after implant insertion. Their study demonstrated a clear increase in the bone density surrounding topical melatonin-associated implants.

Moreover, Melatonin acts by inhibiting bone resorption by suppressing the differentiation and activity of osteoclasts, largely by downregulating the RANKL signalling pathway and increasing the OPG/RANKL ratio [37, 38]. Furthermore, its ability to neutralize reactive oxygen species (ROS) and reduce inflammation at the surgical site creates an optimal microenvironment for new bone formation, leading to enhanced bone density, greater bone-to-implant contact (BIC), and overall higher implant success rates following sinus augmentation [33, 37]. Melatonin has recently been utilized to treat periodontal diseases, reverse bone loss from osteopenia and osteoporosis, and perform bone grafting surgeries [39, 40].

This histomorphometric analysis was helpful in the present study, as it revealed a significant increase in the amount of newly formed bone, by using H&E staining, in the intervention group compared with the control group.

To further evaluate the quality of the newly formed bone, MT staining was used. This type of stain is specific to osteoid tissue (immature) and mature bone tissue. The use of Masson’s Trichrome (MT) to assess bone quality is well-supported in recent literature involving animal bone remodelling highlighting the use of MT staining as a reliable method for differentiating immature osteoid from mature mineralized matrix [41]. The MT-stained sections revealed a significant increase in the percentage of mature bone tissue in the intervention group compared with the control group. Moreover, there was a significant increase in immature bone in the control group compared with the intervention group, indicating that maturation was less active in the control group. The improved bone quality observed in the melatonin group is consistent with recent research findings in which they showed that melatonin plays a key role in upregulating osteogenic markers as Runx2 and Osteocalcin, which aids to prompt angiogenesis and inhibit RANKL mediated bone resorption. This synergistic effect results in faster bone remodelling with melatonin addition as seen in our Masson’s Trichrome histological analysis [42, 43].

Another interesting result was the reduced residual graft percentage observed in the melatonin group. In the current study, the control group exhibited a significantly higher area percentage of residual graft material compared to the study group. This observation is primarily attributed to the baseline composition of the graft; the control group received a higher initial volume of xenograft, which is well-documented for its low substitution rate and long-term persistence in the healing site. While xenografts provide a stable scaffold, their slow resorption means they occupy space that could otherwise be inhabited by vital bone. In contrast, the ability of melatonin to promote osteogenic differentiation and regulate key signaling pathways involved in bone remodelling may explain this result. the integration of melatonin in the study group showed to play a role as a bioactive catalyst rather than a passive filler by significantly accelerating the cellular maturation process required for bone regeneration. This fact is well documented as it acted to reduce the maturation of pre-osteoblasts from 21 days to just 12 days and upregulating essential bone-forming proteins, including alkaline-phosphatase, osteopontin, and osteocalcin [44]. Additionally, it facilitates a favorable healing environment by acting as a potent antioxidant, scavenging reactive oxygen species (ROS) that would otherwise inhibit osteoblastic differentiation and trigger bone destruction [37].

In an experimental study, Hazzaa et al. studied the use of Melatonin with intra-marrow penetration this combination achieved a 55.5% bone fill at eight weeks, versus the use of grafts alone. The authors highlighted that melatonin addition promoted revascularization and regulated the synthesis of type I collagen, which is vital for bone mineralization and osteoblast attachment [45]. This highlights the fact that melatonin can catalyze the change of the graft into functional, living bone rather than just static scaffolding [45]. Another experimental evidence has demonstrated that melatonin enhances mesenchymal stem cell proliferation and osteogenic differentiation through modulation of COX-2/NF-κB and p38/ERK MAPK signaling pathways, resulting in increased new bone formation and improved bone microarchitecture in vivo [37].

Many studies have discussed the application of melatonin coatings in dental implants. In 2017 and 2020 [46, 47], studied the impact of melatonin gel application on autogenous bone grafts around immediate and delayed implants, respectively. In the 2017 study, they reported a significant loss of marginal bone in the control group (1.91 mm), which was greater than that in the test group. In the 2020 study, the authors reported similar results and reported that the combined use of autogenous bone grafts with melatonin is a promising alternative for augmenting early loaded dental implants.

In the present study, the statistical analysis of the radiographic findings after 8 months revealed a significant increase in bone height compared with the preoperative values in both groups. On the other hand, when comparing the crestal bone heights radiographically between the two groups, the results indicated that after 8 months, the bone heights gained in the control group were significantly greater than those in the intervention group. This difference may be due to the anatomical variations which may influence the degree of sinus membrane elevation as well as the amount of graft material placed, in addition to the differences in baseline alveolar bone height among cases. This suggests that the beneficial effects of melatonin may be primarily qualitative rather than volumetric. This finding leads us to hypothesize that melatonin’s mechanism of action preferentially targets the later stages of bone remodelling and maturation, potentially at the expense of the initial, rapid proliferative phase of vertical bone fill. This dissociation highlights the complexity of bone regeneration and underscores the need for future studies to investigate the dose-dependent and time-dependent effects of melatonin on both bone quality and quantity.

The observed findings may be partly explained by the behaviour of conventional inorganic bone graft substitutes, which exhibit slow resorption rates and therefore act as permanent or semi-permanent scaffolds that help preserve vertical ridge dimensions over time [48]. However, this volumetric stability may be associated with biological limitations, as the persistence of non-resorbed graft material can restrict the available space for the formation of vital, well-vascularized host bone [49]. From a clinical perspective, these results suggest that while the control group may favour the maintenance of vertical ridge contour, the conventional treatment appears to promote superior bone quality in terms of density and vascularity. This distinction may be particularly relevant in clinical scenarios where implant stability is a priority, such as in the posterior maxilla characterized by low bone density, although long-term clinical outcomes remain to be validated.

Our findings align with the growing body of evidence supporting various regenerative approaches for the management of the atrophic maxilla. For instance, Cosola et al. (2022) reported consistent radiographic and histomorphological evidence of new bone formation after crestal mini-sinus lift procedures using absorbable collagen [50]. Crespi et al. demonstrated stable long-term radiographic outcomes and favourable bone remodelling following the split-crest technique with immediate implant placement, emphasizing the importance of bone quality assessment over time [51]. Although these surgical approaches differ from the present study, both highlight the critical role of biomaterial selection and biological modifiers in enhancing bone regeneration and implant stability. In this context, the current study contributes novel evidence regarding the potential of melatonin as a biological enhancer in sinus floor elevation, promoting bone maturation and quality rather than merely vertical bone gain.

These results agree with those of Hatem et al. [52], who reported no noticeable difference between the melatonin and graftless groups in terms of implant stability during various follow-up periods or the amount of sinus floor elevation as determined by implant protrusion. However, because melatonin has a physiological effect on bone, the CBCT measurement of relative bone density after nine months revealed a significant difference between the melatonin group and the graftless group in the same study, which was confirmed in our study via histological evaluation [52].

Even though the quantification of inflammatory cells was not performed, the clinical absence of inflammation in both groups highlighted that melatonin acted as an anti-inflammatory at the molecular level in a manner which optimized the microenvironment needed for the regeneration to smoothly happen by down-regulating oxidative stress markers and balance the cytokine profile during the early stages of healing [53].

In agreement with the current radiographic findings, Gendi et al. [34] reported insignificant differences in bone height between their study groups. They examined the effects of melatonin and a hyaluronic acid mixture without a bone graft on bone healing after sinus lifting via a lateral approach with simultaneous implant placement. However, they demonstrated the superior effect of both materials on bone density, which significantly increased in their study group over the follow-up periods of one month and six months after surgery.

Within the limitations of the present study, the use of melatonin gel in lateral maxillary sinus augmentation appeared to enhance bone quality and promote newly formed bone, with a measurable but not superior increase in bone height compared with the control group. Although the sample size was limited due to patient availability and the cost associated with surgical procedures and histological processing, a post-hoc power analysis demonstrated a statistical power of 98.7%, suggesting that the study was adequately powered to detect the observed differences in mature bone formation. The absence of immediate postoperative CBCT imaging, while this prevents the assessment of initial graft volume and its subsequent resorption rate, the study focuses on the final vertical bone height available for implant placement and the quality of the newly formed bone, both of which are critical parameters for the clinical success of a staged sinus lift. Additionally, the evaluated outcomes represent surrogate markers of bone regeneration and do not assess long-term clinical performance, including implant stability, marginal bone loss, or implant survival after functional loading. The current protocol was designed to evaluate the healing period following sinus augmentation and was concluded at this stage. Therefore, larger, adequately powered randomized controlled trials with long-term follow-up are necessary to clarify the volumetric effects of melatonin and its clinical relevance. However, the improvement in bone quality observed histologically in the melatonin group may suggest a positive influence on subsequent implant osseointegration, which warrants further research.

Future research should focus on adequately powered randomized controlled trials designed to evaluate both qualitative and quantitative outcomes of bone regeneration in large maxillary defects. In particular, future studies investigating volumetric bone gain should employ larger sample sizes powered specifically to detect clinically meaningful differences in radiographic bone height. Long-term follow-up studies on the current and future cohorts are also warranted to assess post-loading clinical outcomes, including implant stability at placement and loading, peri-implant marginal bone loss over a minimum follow-up period of 3–5 years after prosthetic loading, and implant survival and success rates. Such investigations are necessary to determine whether the histological improvements observed with melatonin application translate into sustained clinical benefits in implant therapy.

## Conclusion

In conclusion, the present study suggests that melatonin may promote bone maturation and improve histological quality, as evidenced by; higher proportions of newly formed bone, higher proportion of enhanced bone marrow quality, reduced residual bone graft amount applied in sinus augmentation prior to implant placement. However, its effect on radiographic bone height was not significant, possibly due to clinical variations in the degree of sinus membrane elevation and baseline alveolar bone height. This emphasizes the clinical relevance of improving bone quality rather than merely increasing bone height, as higher-quality regenerated bone may contribute to better implant stability and long-term success.

Within the limitations of the current study, melatonin gel shows promise as a biologically supportive adjunct, yet further long-term clinical trials with larger cohorts are required to validate its clinical significance in maxillary sinus augmentation.

## Supplementary Information

Supplementary Material 1.

Supplementary Material 2.

Supplementary Material 3.

## Data Availability

The datasets used and/or analyzed during the current study are available from the corresponding author upon request.

## Abbreviations

RANKL: Receptor Activator of Nuclear Factor Kappa-B Ligand
ORN: Osteoradionecrosis
BMP-2: Bone Morphogenic Protein-2
ONC: Osteonectin
ALP: Alkaline Phosphatase
TRAP: Tartrate-Resistant Acid Phosphatase
RCTs: Randomized Controlled Trials
CBCT: Cone Beam Computed Tomography
MLN: Melatonin
RPM: Round per minute
DBBM: Deproteinized Bovine Bone Mineral
H&E: Hematoxylin and Eosin
MT: Masson’s trichrome
Mm: Millimetre
