# Supplementary figures and images for: Clinical, radiographic and histomorphometric assessment of the effects of melatonin gel mixed with a xenograft in augmentation of the maxillary sinus: a randomized controlled clinical trial
Source: BMC Oral Health. 2026 Apr 16;26:732. doi: 10.1186/s12903-026-08201-0 (PMC13110429; doi:10.1186/s12903-026-08201-0)

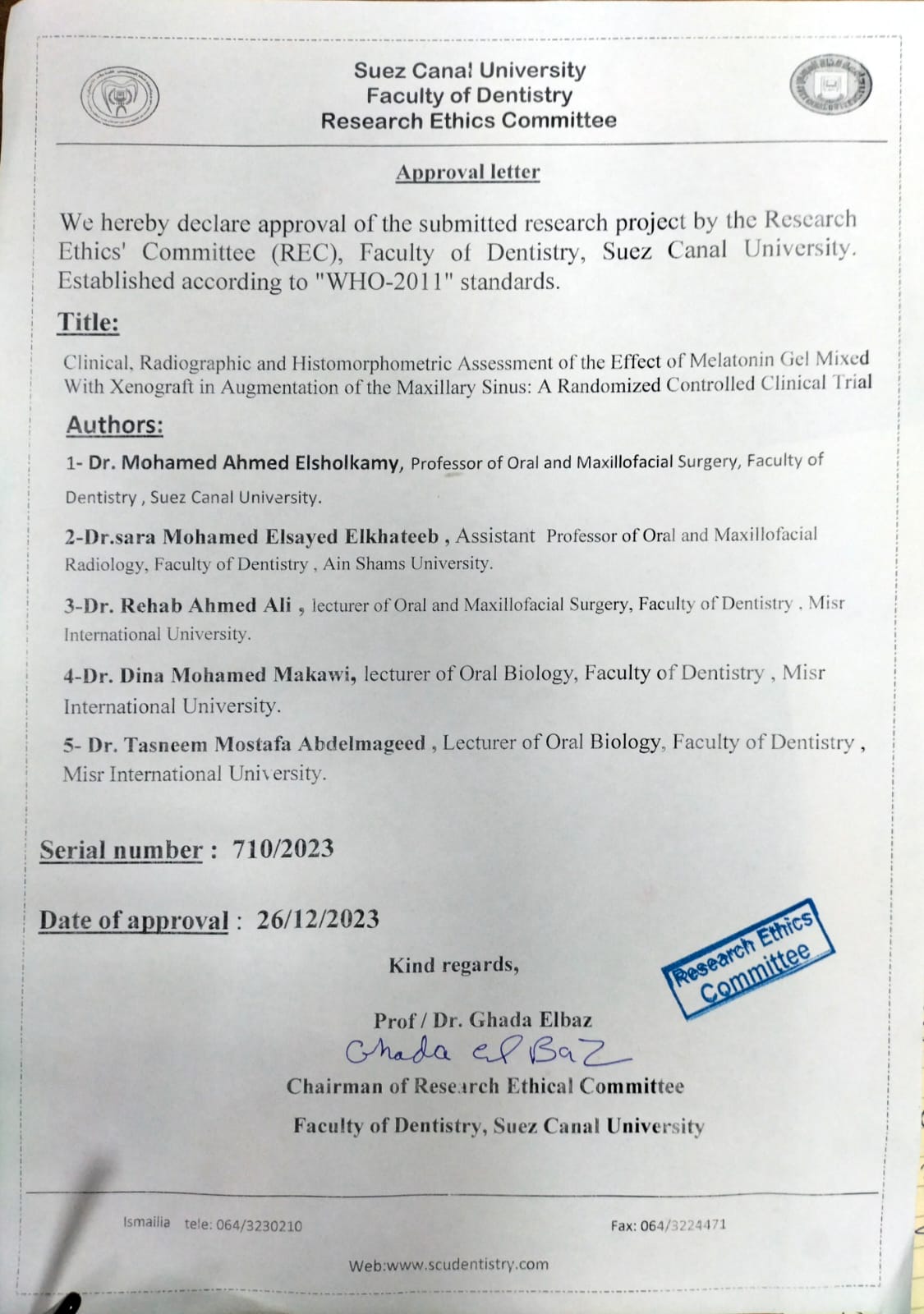

Supplement: Supplementary file 1 — Supplementary Material 1. [file 12903_2026_8201_MOESM1_ESM.jpeg]
