# Supplementary material for: Clinical, radiographic and histomorphometric assessment of the effects of melatonin gel mixed with a xenograft in augmentation of the maxillary sinus: a randomized controlled clinical trial
Source: BMC Oral Health. 2026 Apr 16;26:732. doi: 10.1186/s12903-026-08201-0 (PMC13110429; doi:10.1186/s12903-026-08201-0)
Supplement: Supplementary file 2 — Supplementary Material 2. [file 12903_2026_8201_MOESM2_ESM.pdf]

ClinicalTrials.gov PRS DRAFT Receipt (Working Version)
Last Update: 12/14/2024 08:19

ClinicalTrials.gov ID: NCT06736821

Study Identification

Unique Protocol ID: 710/2023

Brief Title: Maxillary Sinus Augmentation by Xenograft Mixed With Melatonin (melatonin)

Official Title: Clinical, Radiographic and Histomorphometric Assessment of the Effect of Melatonin Gel Mixed With Xenograft in Augmentation of the Maxillary Sinus: A Randomized Controlled Clinical Trial

Secondary IDs:

Study Status

Record Verification: December 2024

Overall Status: Completed

Study Start: January 9, 2024 [Actual]

Primary Completion: July 23, 2024 [Actual]

Study Completion: August 27, 2024 [Actual]

Sponsor/Collaborators

Sponsor: rehab soliman

Responsible Party: Sponsor-Investigator
Investigator: rehab soliman [rehab soliman]
Official Title: lecturer of oral and maxillofacial department
Affiliation: Ain Shams University

Collaborators:

Oversight

U.S. FDA-regulated Drug: No

U.S. FDA-regulated Device: No

U.S. FDA IND/IDE: No

Human Subjects Review: Board Status: Approved
Approval Number: 710/2023
Board Name: research ethical committee
Board Affiliation: suez canal university faculty of dentistry
Phone: 0643230210
Email: president_office@suez.canal.eg
Address:

4.5km ring road, Ismailia, Egypt

Data Monitoring: No

FDA Regulated Intervention: No

Study Description

Brief Summary: The possibility of enhancing the bone substitute in the maxillary sinus by mixing it with the melatonin to allow proper implant placement in early stage .

The aim of the study was to evaluate clinically, radiographically and histologically the effect of melatonin in enhancement of bone healing after augmentation of the maxillary sinus.

Detailed Description: This study was include 16 severely atrophied maxillary sinus, with alveolar bone height less than 4mm, that need sinus augmentation and delayed implant placement, they were be equally and randomly distributed into 2 groups (via : http://www.randomizer.org) :

Group A: sinuses was received sinus membrane elevation using lateral window technique and filling the sinus with the mixture of melatonin and xenograft.

Group B: sinuses was received sinus membrane elevation using lateral window technique and filling the sinus with xenograft.

After 6 months, bone biopsies was collected from the augmented sinuses immediately before implant instalment in the same visit.

Initial cone beam computerized tomography was taken preoperatively and immediate postoperatively.

Final cone beam computerized tomography was taken 6 months before implant placement to measure Bone density and bone height and width.

After 3 months of implant placement, the installed implants was assessed clinically to exclude any infection or dehiscence and radio graphically to assess the bone loss or any radiolucent lesion around the implants and then prepared for loading.

Conditions

Conditions: Bone Healing

Keywords: sinus lifting
maxillary sinus augmentation
melatonin gel

Study Design

Study Type: Interventional

Primary Purpose: Treatment

Study Phase: N/A

Interventional Study Model: Parallel Assignment
This study was included16 severely atrophied maxillary sinus, with alveolar bone height less than 4mm, that need sinus augmentation and delayed implant placement. all patients was enrolled randomly and distributed into 2 groups (via : http://www.randomizer.org) :

	Group A: sinus membrane elevation was done using lateral window technique and filled with the mixture of melatonin and xenograft.<br><br>Group B: sinuses membrane was elevated and filled with with xenograft. After 6 months, bone biopsies was collected from the augmented sinuses immediately before implant instalment in the same visit.<br><br>Initial CBCT was taken preoperatively and immediate postoperatively. Final CBCT was taken 6 months before implant placement to measure Bone density and bone height and width.
Number of Arms:	2
Masking:	Triple (Participant, Investigator, Outcomes Assessor)
Allocation:	Randomized
Enrollment:	16 [Actual]

Arms and Interventions

Arms	Assigned Interventions
Experimental: group a: xenograft mixed with melatonin<br>in this group, the patients underwent open sinus lifting surgery and augmentation of the sinuses by xenograft mixed with melatonin gel to enhance the bone healing	Dietary Supplement: Open sinus augmentation with melatonin gel mixed with xenograft<br>All Patients in this group underwent open sinus procedures to lift the sinus membrane followed by application of the melatonin gel which was mixed with xenograft as an augmentation material. Preclinical studies proved the direct action of melatonin in enhancing the differentiation and proliferation of bone-forming osteoblasts. In addition to increasing bone mass, melatonin also facilitates new bone growth and osteointegration, making melatonin a particularly attractive molecule for use in bone implants when used alone or in combination with other growth factors<br>Other Names:<br>• Primary intervention
Active Comparator: group b: xenograft<br>in this group, the patients underwent open sinus lifting surgery and augmentation of the sinuses by xenograft without any additives	Procedure/Surgery: Open sinus augmentation with xenograft<br>All Patients in this group underwent open sinus procedures to lift the sinus membrane followed by application of the xenograft as an augmentation material xenograft is used instead of autogenous bone graft to avoid the morbidity of the donor side<br>Other Names:<br>• Secondary Intervention

Outcome Measures

Primary Outcome Measure:

1. bone height
   will be measured using CBCT (Cone Beam Computed Tomography)

   [Time Frame: 6 months]

Secondary Outcome Measure:

2. histological analysis

will be measured using hematoxylin and eosin stains and messon trichrome stain.

[Time Frame: 6 months]

Eligibility

Minimum Age: 25 Years

Maximum Age:

Sex: All

Gender Based:

Accepts Healthy Volunteers: Yes

Criteria: Inclusion Criteria:

1. Adult male/female patients above the age of 25.
2. Patients with one or more teeth requiring implant supported dental restoration in atrophic maxilla (unilateral or bilateral).
3. Alveolar bone height less than 4 mm at the defective site.
4. Good oral hygiene.

Exclusion Criteria:

1. Medically compromised patients with conditions contraindicating surgery (eg. uncontrolled diabetics, bisphosphonate intake, radio or chemotherapy).
2. Patients with active infection at or related to the site of surgery (eg. acute sinusitis).
3. Heavy smokers.
4. Patients not indicated for an implant supported restoration at the time of enrollment (eg. active/untreated periodontal disease).

Contacts/Locations

Central Contact Person: rehab a soliman, lecturer
Telephone: +201018662326
Email: rehabsoliman4@gmail.com

Central Contact Backup: tasneem soliman

Study Officials: NOTE : Study Official is required by the WHO and ICMJE.

Locations: **Egypt**
suez canal university
Ismailia, Egypt, 41522
Contact: rehab a soliman, lecturer +201018662326 rehabsoliman4@gmail.com
Contact: tasneem soliman, lecturer +20127189914 tassnim.mostafa@miuegypt.edu.eg
Principal Investigator: Mohamed ElSholkamy El Sholkamy, professor
Sub-Investigator: sara elsayed elkhateeb, associate professor
Sub-Investigator: dina mohamed makawi, lecturer
Sub-Investigator: tasneem soliman, lecturer
Principal Investigator: rehab ali soliman, lecturer
Sub-Investigator: sarah almugairn, associate professor

NOTE : Locations have 6 notes.

IPDSharing

Plan to Share IPD: No

References

Citations:

Links:

Available IPD/Information:
